# Oral Activated Charcoal Prevents Experimental Cerebral Malaria in Mice and in a Randomized Controlled Clinical Trial in Man Did Not Interfere with the Pharmacokinetics of Parenteral Artesunate

**DOI:** 10.1371/journal.pone.0009867

**Published:** 2010-04-15

**Authors:** J. Brian de Souza, Uduak Okomo, Neal D. Alexander, Naveed Aziz, Benjamin M. J. Owens, Harparkash Kaur, Momodou Jasseh, Sant Muangnoicharoen, Percy F. Sumariwalla, David C. Warhurst, Stephen A. Ward, David J. Conway, Luis Ulloa, Kevin J. Tracey, Brian M. J. Foxwell, Paul M. Kaye, Michael Walther

**Affiliations:** 1 Department of Infectious and Tropical Diseases, London School of Hygiene and Tropical Medicine, London, United Kingdom; 2 Division of Infection and Immunity, Department of Immunology, University College London, London, United Kingdom; 3 Medical Research Council Laboratories, Fajara, Banjul, The Gambia; 4 The Technology Facility, Department of Biology, University of York, York, United Kingdom; 5 Centre for Immunology and Infection, Hull York Medical School and Department of Biology, University of York, York, United Kingdom; 6 Liverpool School of Tropical Medicine, Liverpool, United Kingdom; 7 Kennedy Institute of Rheumatology, Imperial College of Science, London, United Kingdom; 8 Center of Immunology and Inflammation, North Shore-LIJ Research Institute, New York, New York, United States of America; The George Washington University Medical Center, United States of America

## Abstract

**Background:**

Safe, cheap and effective adjunct therapies preventing the development of, or reducing the mortality from, severe malaria could have considerable and rapid public health impact. Oral activated charcoal (oAC) is a safe and well tolerated treatment for acute poisoning, more recently shown to have significant immunomodulatory effects in man. In preparation for possible efficacy trials in human malaria, we sought to determine whether oAC would i) reduce mortality due to experimental cerebral malaria (ECM) in mice, ii) modulate immune and inflammatory responses associated with ECM, and iii) affect the pharmacokinetics of parenteral artesunate in human volunteers.

**Methods/Principal Findings:**

We found that oAC provided significant protection against *P. berghei* ANKA-induced ECM, increasing overall survival time compared to untreated mice (p<0.0001; hazard ratio 16.4; 95% CI 6.73 to 40.1). Protection from ECM by oAC was associated with reduced numbers of splenic TNF^+^ CD4^+^ T cells and multifunctional IFNγ^+^TNF^+^ CD4^+^ and CD8^+^ T cells. Furthermore, we identified a whole blood gene expression signature (68 genes) associated with protection from ECM. To evaluate whether oAC might affect current best available anti-malarial treatment, we conducted a randomized controlled open label trial in 52 human volunteers (ISRCTN NR. 64793756), administering artesunate (AS) in the presence or absence of oAC. We demonstrated that co-administration of oAC was safe and well-tolerated. In the 26 subjects further analyzed, we found no interference with the pharmacokinetics of parenteral AS or its pharmacologically active metabolite dihydroartemisinin.

**Conclusions/Significance:**

oAC protects against ECM in mice, and does not interfere with the pharmacokinetics of parenteral artesunate. If future studies succeed in establishing the efficacy of oAC in human malaria, then the characteristics of being inexpensive, well-tolerated at high doses and requiring no sophisticated storage would make oAC a relevant candidate for adjunct therapy to reduce mortality from severe malaria, or for immediate treatment of suspected severe malaria in a rural setting.

**Trial Registration:**

Controlled-Trials.com ISRCTN64793756

## Introduction

Severe malaria encompasses a broad range of clinical syndromes resulting mainly from infection with *Plasmodium falciparum*, and is estimated to be responsible for the death of 0.5–1.0 million African children every year [1]. In addition to the neurological syndrome of cerebral malaria (CM), death from severe malaria in children may result from severe anaemia and/or respiratory distress leading to metabolic acidosis. Although the pathophysiologic basis of severe malaria is complex and may likely include reduced deformability of [2], [3] as well as cytoadherence of [4]–[6] parasitized red blood cells, convincing arguments have nevertheless been made for an important role of pro-inflammatory cytokines in many aspects of disease [7]–[9]. Failure to break the vicious cycle of metabolic changes induced by excess cytokine production contributes significantly to the high mortality rates observed. However, attempts to improve survival by targeting individual cytokines, notably TNF, have been largely unsuccessful [10]. Thus, we examined alternate approaches to overcome the detrimental effects of this cytokine cascade, with a view to identifying an intervention that was appropriate for use in resource-poor countries and that, given the urgent clinical need, might be rapidly available for clinical use.

Three observations led us to examine a potential role for activated charcoal (AC) in the treatment of severe malaria. First, a number of studies have demonstrated that in *ex vivo* haemofiltration, AC is highly effective at adsorbing a range of endotoxin-induced cytokines from the bloodstream, including TNF, IL-1 and IL-6 [11]. Second, TNF-dependent lethality in models of endotoxemia is associated with delivery of TNF to the intestinal lumen via the bile duct. Thus, bile duct cannulation protects rats from lethal endotoxemia [12] (Ulloa et.al. unpublished), raising the possibility that AC in the intestinal lumen might directly affect cytokine availability. Third, oAC has for many years been used in the clinic to suppress chronic kidney disease, through indirect modulation of inflammation [13].

We now report in this study that oral administration of AC (oAC) close to the time of onset of experimental CM (ECM) protects mice from death and extends overall survival time even in the face of high parasitemia. Analysis of splenic T cell cytokine production found that oAC treatment was associated with reduced numbers of CD4+ and CD8+ T cells committed to produce TNF and IFNγ, and gene expression profiling identified a whole blood ‘signature’ associated with oAC-mediated protection from ECM. Based on these promising results, we conducted a clinical trial to evaluate whether oAC would alter the pharmacokinetics of parenteral artesunate (AS), currently the most efficient chemotherapy for severe malaria [14], and found that oAC had little impact on the area under the curve (AUC) or the serum half life of either AS or its active metabolite, dihydroartemisinin (DHA). Together, these data encourage future studies to evaluate the potential efficacy of oAC in human malaria.

## Materials and Methods

### Mice, Parasites and establishment of ECM

C57BL/6 mice were purchased from Harlan and were housed under barrier conditions at the London School of Hygiene and Tropical Medicine. Mice used in all experiments were sex-matched and used at 6 wk of age. *P. berghei* ANKA (PbA) was originally obtained from Dr. N. Wedderburn (Royal College of Surgeons, London, UK) and used in all experiments after one in vivo passage in C57BL/6 mice. Mice were infected intravenously with 10^4^ parasitized red blood cells (pRBC). Actidose-Aqua® activated charcoal (0.2g charcoal/ml) was obtained from Paddock laboratories, Inc. (Cat# NDC0574-0121-04), and at day 3 and day 5 post infection (p.i.) mice were randomized to receive 130 mg charcoal/kg (administered orally in 100 ul volume saline), based on initial dose titration studies in a model of endotoxemia (Ulloa et. al., unpublished) or saline alone. In some studies, Actidose-Aqua® charcoal was used after washing into sterile physiological saline, or an alternate source of activated granulated charcoal was used (Aktivkohle, Granulat, 1.5 mm, reinst by Caesar & Loretz GmbH, Germany). Mice were not anesthetized or sedated during dosing as this frequently resulted in airway contamination. Mice were monitored for neurological signs of CM, including convulsions, ataxia and paralysis at frequent intervals daily. Parasite burden was determined from Giemsa stained blood smears, and expressed as the percentage of pRBCs. RBCs were counted using a haemocytometer by diluting 2 µl of tail blood in 1 ml RPMI. Animals were monitored every evening and killed by cervical dislocation when death was deemed inevitable before the next morning, according to UK Home Office guidelines (The Animals (Scientific Procedures) Act 1986). Blood was harvested by cardiac puncture for isolation of PBMC. Brain tissue was carefully removed and fixed in 4% formol saline for wax embedding, and preparing tissue sections for hematoxylin and eosin staining.

### Cytokine production by splenic T cells

Spleens were isolated from uninfected mice, PbA-infected mice and PbA-infected, AC-treated mice and single cell suspensions generated by passing tissue through a 100 µm cell strainer. Erythrocytes were lysed using Gey's solution and cells were washed twice in complete RPMI-1640 (RPMI supplemented with 2 mM L-glutamine, 100 U/ml penicillin and 100 µg/ml streptomycin, plus 5% Foetal calf serum). Cells were either i) cultured directly ex vivo in 10 µg/ml Brefeldin A (Sigma-Aldrich, UK) for 4 hours or ii) restimulated for 2 hours by incubation with 10 ng/ml PMA (Sigma-Aldrich, UK) and 1 µg/ml Ionomycin (Sigma-Aldrich, UK) before addition of Brefeldin A. After incubation, cells were washed in PBS, 5 mM EDTA, 2% FCS and labeled with phycoerythrin (PE)-Cy7 conjugated anti-CD3ε (145-2C11, eBioscience, UK), PerCP conjugated anti-CD4 (RM4-5) and allophycocyanin (APC) conjugated anti-CD8α (53-6.7, both from BD Pharmingen, San Diego, USA) for 30 minutes on ice. After labeling cells were washed twice in PBS, 5 mM EDTA, 2% FCS and fixed in paraformaldehyde. Cells were permeabilised by washing in PBS containing 0.5% bovine serum albumin and 0.5% saponin followed by labeling with combinations of Pacific Blue conjugated anti- IFN-γ (XMG1.2) and PE conjugated anti-TNF-α (MP5-XT22), or appropriate isotype controls (all from eBioscience, UK) for 45 minutes on ice. After labeling, cells were washed in saponin containing buffer and twice in saponin-free buffer. Flow cytometric analysis was performed on >100, 000 cells in a CyAn ADP flow cytometer with Summit analysis software (Beckman Coulter).

### Whole blood gene expression profiling

A group of 20 female C57BL/6 mice were infected with PbA and at day 3 and 5, 10 mice received oAC, as above. At day 6 p.i., and prior to the first deaths of untreated PbA-infected mice, 5 untreated PbA-infected mice (Group 1) and 5 oAC-treated PbA-infected mice (Group 2) were killed and fresh blood (300–500 µl per mouse) was collected and processed into RNA using a Mouse RiboPure™-Blood RNA isolation Kit (Ambion), according to the manufacturer's instructions. Blood was also taken from uninfected control mice (‘baseline’). The remaining infected mice in each group were then followed for the development of ECM. Total RNA was isolated according to the manufacturer's protocol. RNA concentration and integrity was established using a 210 Bioanalyser (Agilent Technologies, Palo Alto, CA). RNA samples from each mouse were treated independently. Extracted RNA was reverse transcribed to cDNA using the Affymetrix GeneChip one-cycle target labelling kit (Affymetrix, Santa Clara, CA) according to the manufacturer's recommended protocols and hybridised to GeneChip® Mouse 430 2.0 Genome Array. Raw data processing was performed by using the Affymetrix GCOS 1.2 software. After hybridization and scanning, probe cell intensities were calculated and summarized for the respective probe sets by means of the MAS5 algorithm. MAS5 normalised data were collected and analyzed by using the ArrayAssist Expression software, Version 5.5 (Stratagene). Raw data derived from individual whole blood gene expression profiles were filtered for expression level, discarding the lowest 20^th^ percentile that represented non-expressed genes, producing a 39,299 gene list. This list was first filtered for genes that had a P value corrected by false discovery rate (Benjamini and Hochberg False Discovery Rate) of less than 0.05 and then by specifying at least 2-fold difference in expression between any of the three groups. The 12,365 gene list generated in this way was further filtered for genes differentially expressed only between oAC-treated and untreated PbA-infected mice, generating a list of 99 genes. This was filtered further manually to removal duplicates and probes with unpredictable levels of cross-hybridization, and generated a 68 gene list. Hierarchical clustering (using a Euclidean distance metric and a centroid linkage matrix) was performed in GeneSpring v7.3.1, allowing construction of a heat map that displayed both a sample tree and a gene tree. Data from these studies have been deposited in the EBI ArrayExpress data base (Accession #: E-MEXP-2594).

### Data handling and Statistical analysis – mouse models

For assessing the impact of oAC, results represent pooled data derived from 4 independent experiments involving a total of 23 control and 31 oAC-treated mice. Generation of survival curves, log rank tests and the calculation of hazard ratios were all performed in Prism v5.01 (GraphPad software Inc). For cytokine analysis, data are pooled from two independent experiments analyzed (n = 10 mice per treatment group and 10 control uninfected mice). Data were analyzed using a Kruskal-Wallis non parametric ANOVA (p<0.0001 for all comparisons), with Dunn's post test used to compare results for each cytokine. To compare global gene expression profiles in mice with and without treatment, three cohorts of 10 mice (uninfected ‘baseline’, infected and treated, infected and not treated) were used. 5 mice per treatment group were killed at d5 for gene expression analysis and 5 mice were allowed to proceed to develop ECM. Fisher's Exact Test was used to analyze protection from ECM at day 9. Data from microarray analysis was analyzed as detailed above.

### Open-label Phase I trial: Study population and design

The pharmacokinetic study was conducted as an open label randomized controlled trial at the Armed Forces Provisional Ruling Council (AFPRC) General Hospital in Farafenni, the Gambia from February to June 2007 according to ICH/GCP guidelines, and independently monitored by the MRC Gambia Unit Clinical Trials Support Manager. The primary objective was to evaluate whether oral AC altered the pharmacokinetics of intravenously applied AS and its metabolite dihydroartemisinin (DHA), when given simultaneously or after 1 hour. The protocol for this trial and supporting CONSORT checklist are available as supporting information; see Checklist S1 and Protocol S1.

After initial sensitisation visits to 18 villages all adult (aged 21–45 years) inhabitants were invited for meetings, during which the details of the study were explained and the information sheet made available to those interested in the study. A total of 187 volunteered for the screening for which oral consent was obtained, and were examined for malaria parasitaemia (OptiMAL- IT**®** test, DiaMed AG, Cressier, Switzerland) and anaemia (Hemocue photometer, Hemocue, Angelholm, Sweden). Upon presentation at the hospital, parasite negative status was confirmed again by slide microscopy and pregnancy was ruled out using a urine dip stick. Fifty-two healthy adults (negative for malaria, Hb >11 g/dl, no reported drug intake during the previous week, not pregnant), were enrolled after written informed consent was obtained, and hospitalized to the research ward for 24 hours. To ensure equal numbers per group after every 15 participants, volunteers were block-randomised to one of three study arms, using random numbers generated by the random numbers generation function from Excel, Microsoft. In order of appearance at the random numbers generator, these numbers were allocated to group 1, 2 or 3, respectively by the PI, so that after each set of 15 numbers an equal distribution to all three groups is guaranteed. Cards labelled with the group numbers 1 to 3 were then put into envelopes labelled with the numbers that have been randomized to each group. The sealed envelopes were then arranged in ascending order, and were allocated one after the other by the study physician to eligible subjects in the order they were enrolled in the study. The study consisted of the following arms: arm 1 (control), receiving i.v. AS and water; arm 2, receiving i.v. AS and oral AC simultaneously; and arm 3, receiving i.v. AS and oral AC 1 h later. Each treatment schedule was administered twice to each individual, starting on admission and 12 h thereafter.

A clinical assessment, full blood count (FBC), and routine biochemistry were performed on all participants. AS in bicarbonate (Guilin pharma) was given to all study participants intravenously at a dose of 2.4 mg/kg bodyweight at 0 hours, and after 12 hours. 50 g of AC (Aktivkohle, Granulat, 1.5 mm; Caesar & Loretz GmbH, Germany) in 350 mls of water were given orally. The clinical assessment was repeated prior to administering the second dose of drugs. Blood samples were taken 5, 10, 15, 30, 60 and 90 min, and 3 h and 6 h after the second dose of AS (at 12 h) and centrifuged immediately (420 g, 10 min at ambient temperature). Plasma was snap-frozen in liquid nitrogen without delay. Prior to discharge routine biochemistry and clinical assessment were repeated.

### Measurement of AS and DHA

The trial endpoint was to measure the plasma levels of AS and its metabolite DHA by High Performance Liquid Chromatography (HPLC) - Mass Spectrometry (MS, Thermo Acella HPLC and Thermo Quantum Access Mass Spectrometer) after the second dose of artesunate had been given (at 12 hours), and to determine their C_max_, t_max_, t_½_ and AUC. The MS was operated in positive heated electrospray ionization mode with spray voltage 4.5 KV, and capillary temperature 250°C. The mobile phase comprising of 60% acetonitrile and 40% 0.1 M ammonium acetate was run for 4 min at a flow rate 450 uL per minute. AS and DHA were separated using a Thermo Betasyl Phenyl Hexyl 2.1×50 mm HPLC column connected with a guard column of the same packing material. Artemisinin was used as internal standard. Assays were validated from 1–2000 ng/mL. All samples were coded with a three digit identifier, and investigators in the laboratory were blinded to the group assignment of each volunteer.

### Data handling and Statistical analysis: clinical data

Data were double entered into an Access database. The primary endpoint was the area under each subject's concentration-time curve (AUC) for AS and DHA. Single compartment pharmacokinetic models were fitted for each person by least squares, using the nlminb function of S-Plus®. For some subjects, the one-compartment model for AS levels yielded very highly correlated parameter estimates, probably because the sampling scheme did not track the very rapid initial rise in AS plasma levels. For this reason, a simple exponential model was also fitted to each subject's AS data, and compared by analysis of variance (ANOVA) to the single compartment model. Parameter estimates for AS were taken from the single compartment model if the p value was less than 0.1, otherwise from the simple exponential. AUC was estimated as the dose divided by CL/F, where CL is clearance and F is bioavailability. Since F is unknown, CL/F is estimated as a single parameter. AUC and other pharmacokinetic parameter estimates were compared between the trial arms by ANOVA. Confidence intervals for the between-arm differences in geometric means of the parameter estimates were obtained by contrasts [15].

To achieve 80% power to detect a ratio of means of 1.5 of mean AUC, or other pharmacokinetic parameters, between control and either intervention arm, with coefficient of variation of 50% and two-sided significance level of 5%, we calculated that 23 subjects per arm would be necessary [16]. We were able to enrol 52 individuals (75% of the calculated sample size). Due to logistical constraints, samples for 46% of the 52 study participants could only be statistically analysed after 20 months storage. Unfortunately, a rather implausible profile was obtained for DHA in that a rapid decline was observed within 15–30 min, regardless of the study arm, possibly due to the decay of the samples on storage even at −80°C. These data were therefore excluded from further analysis.

### Scientific and Ethical Review

All animal experiments were performed in accordance with UK Home Office regulations, under protocols approved by the LSHTM Animal Ethics Procedures Committee. The clinical study was reviewed and approved by the Scientific Coordinating Committee of the MRC the Gambia, the LSHTM and University of York Ethics Committees, and the Joint Gambian Government/MRC Ethics Committee and registered with ISRCTN (Nr. 64793756).

## Results

### oAC protects mice from cerebral malaria

**Figure 1:**
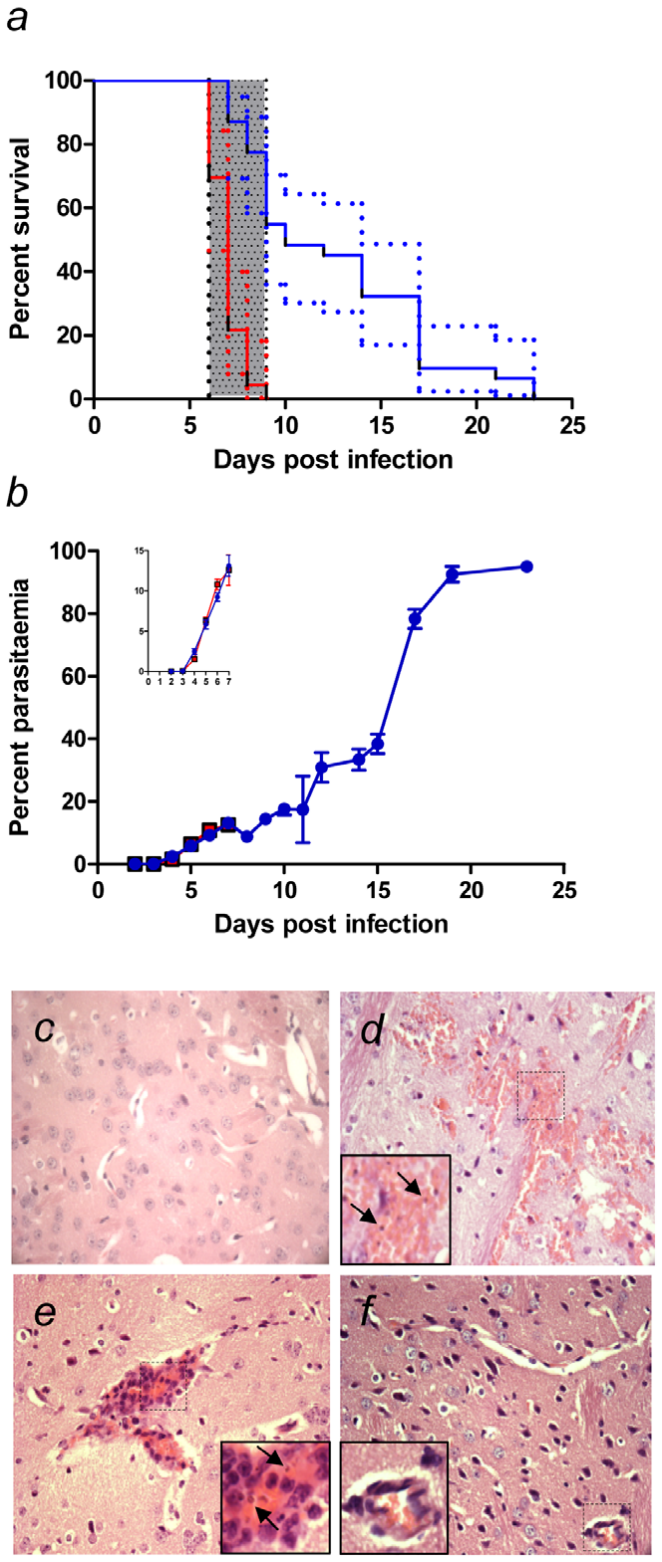
AC treatment for experimental cerebral malaria. **a**) C57BL/6 mice were infected with PbA and either untreated (red line) or treated at d3 and d5 with oAC (Blue line). Survival was monitored over a 25 day period. The window for CM deaths (d6-9) is indicated by the hatched bar. Data are pooled from 5 independent experiments (n =  23 untreated and n =  31 oAC-treated mice) and are shown with 95% CI (dotted lines). Overall survival was significantly improved by oAC treatment (p<0.0001). **b**) Parasitemias in mice infected with PbA (open squares) and in PbA-infected mice treated with oAC (closed squares) are shown. Data represent mean ± SEM. Insert shows parasitemias over d1-7, enlarged for clarity. **c–f**) Brain histopathology of normal mice (c), mice infected for 6 days with PbA (d, e), and mice infected with PbA and treated with oAC (f). In d) and e), inserts show regions of perivascular hemorrhage and parasitized RBC (arrows). H&E staining; original magnification ×40.

To test the hypothesis that oral administration of AC might have a beneficial effect on the outcome of severe malaria, we used the model of ECM caused by *Plasmodium berghei* ANKA (PbA) infection in C57BL/6 mice. This is a well-accepted model for many aspects of human disease; pro-inflammatory cytokines are abundant; mice develop CNS lactic acidosis, increased blood-brain barrier permeability, paralysis, seizures and death; and there are similarities in brain histopathology [17]. All untreated mice infected with PbA developed severe neurological symptoms, including convulsions and ataxia from 5–6 days post-infection (p.i.), and almost invariably died rapidly thereafter (in a time window of 6–9 days. As shown in Fig. 1a, in untreated mice infected with PbA, only 5/23 (21.7%) mice survived past day 7 and 0/23 (0%) survived past day 9). In contrast, mice administered AC (Actidose-Aqua®) by oral gavage on days 3 and 5 p.i. were highly resistant to the development of ECM, with 27/31 (87.1%) surviving past day 7 and 17/31 (54.8%) surviving past day 9 (**Fig. 1a**). As no anti-malarial agents were administered, oAC-treated mice eventually became hyper-parasitemic and died, presumably from anemia. Nevertheless, oAC significantly prolonged overall survival time (Fig. 1*a*; χ^2^_1_ = 37.8, *P<*0.0001; hazard ratio 16.4; 95% CI of ratio: 6.73–40.1). Strikingly, some treated animals survived for long periods despite parasitaemias in excess of 75% (**Fig. 1b**). To confirm that these results were attributable to the charcoal component of Actidose-Aqua® and not some other possible factor in the commercial diluent, we separated the AC and administered this to mice re-suspended in physiological saline, with other mice receiving the original diluent. In this experiment, 5/5 mice treated with diluent alone died from ECM at d7 p.i., whereas 0/5 mice treated with Actidose-Aqua® - derived AC died by day 9 p.i. and 3/5 survived beyond day 10 (χ^2^_1_ = 9; p = 0.003). Next, we separately sourced a stock of AC in granule form and after crushing to a powder, administered this as a suspension in saline. This mixture was also highly effective at protecting against ECM (40% protection vs. saline control, p = 0.002) and had no impact on parasitemia or anemia (data not shown). To evaluate whether the timing of oAC treatment was critical for its effectiveness, we gave oAC in varying schedules. oAC given at days 4, 5 and 6 p.i. was as effective at protecting from ECM as oAC given at days 3 and 5p.i. (p = 0.54 for survival of mice treated with oAC on d3 + 5 vs. d4 + 5 + 6). A single dose of oAC at day 6 p.i. was still sufficient to protect mice from rapidly succumbing to ECM (100% vs. 40% survival at day 7 for oAC-treated and untreated PbA-infected mice, respectively), though ultimately all mice receiving only a single dose of oAC by day 9 died of ECM. We conclude that oAC, while not having a major effect on parasitemia, is highly protective against the development of ECM when given shortly prior to or at the onset of symptoms. The effect of repeated administration of oAC to mice after the onset of symptoms could not be tested due to practical difficulties in oral administration of AC to mice at such times.

To determine whether oAC treatment prevented the brain histopathology associated with CM, brain sections were stained with hematoxylin and eosin. Compared to normal brain (**Fig. 1c**), brains from untreated PbA-infected mice showed evidence of intra-cerebral injury, including peri-vascular haemorrhages containing parasitized red blood cells (**Fig. 1d–e**) as described previously [18], [19]. In addition, many blood vessels were extensively occluded with thrombi composed of parasitized erythrocytes. In contrast, these histological changes were not observed in PbA-infected mice treated with oAC (**Fig. 1f**).

### oAC reduces pro-inflammatory cytokine production by splenic T cells

**Figure 2:**
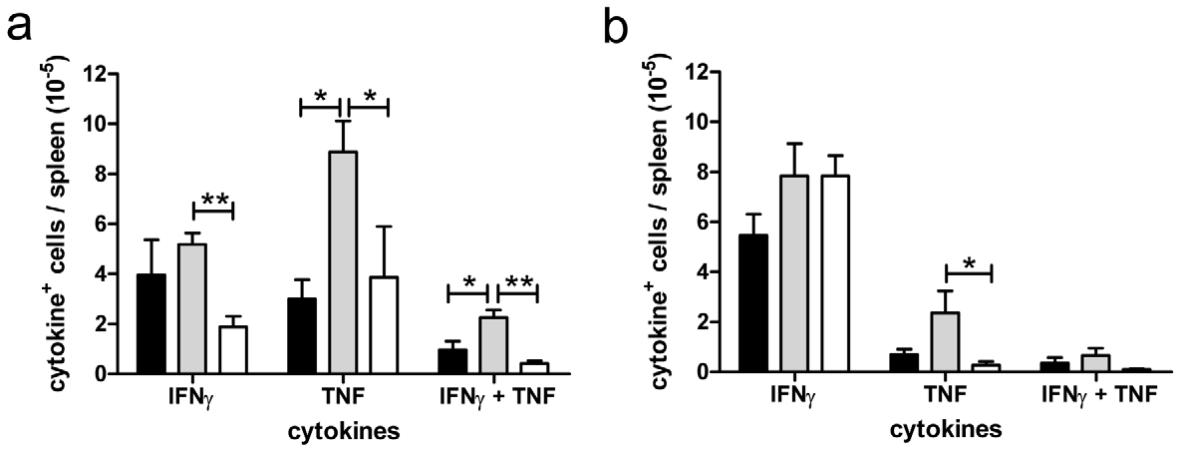
AC treatment affects T cell cytokine production. PbA infected (grey bars) and PbA-infected oAC-treated (black bars) mice were killed on day 6 and cytokine measured after PMA stimulation. The absolute number of splenic IFNγ^+^, TNF^+^ and IFNγ^+^TNF^+^ CD4^+^ (a) and CD8^+^ (b) are shown. Uninfected mice (open bars) are shown as baseline. Data represent mean ± SE (n = 10 individual mice from 2 independent experiments). *, p<0.05; **, p<0.01; ***, p<0.001.

Pro-inflammatory cytokines have a long-standing association with the outcome of experimental and human malaria (reviewed in [20]–[22]). To determine whether oAC affected production of pro-inflammatory cytokines by splenic CD4^+^ and CD8^+^ T cells, we used multi-parameter intracellular flow cytometry. By direct *ex vivo* analysis, no differences were observed between the number of CD4^+^ and CD8^+^ T cells producing TNF, IFNγ or IL-17 (data not shown). However, as direct ex vivo analysis of cytokine production only reveals a snap-shot of T cell function and may not fully reflect the capacity for polyfunctional cytokine responses [23], we also examined the response following 2 h stimulation with PMA (**Fig. 2**). The number of CD4^+^ T cells producing IFNγ alone was largely unaffected by oAC treatment. However, whereas CD4^+^ T cells co-producing IFNγ^+^ and TNF^+^ and those producing TNF alone were clearly increased in frequency in untreated PbA-infected mice compared to uninfected mice, this was not the case in mice treated with oAC (**Fig. 2a**). A similar trend was also observed for CD8^+^ T cells, though this was only significant in the case of non-IFNγ-producing, TNF-producing cells (**Fig. 2b**). CD4^+^ and CD8^+^ T cells producing IL-10 were only detected in a minority of mice irrespective of treatment and at very low abundance (<10^4^ per mouse; data not shown). In summary, oAC treatment resulted in a reduced commitment for pro-inflammatory cytokine production amongst CD4^+^ and CD8^+^ T cells when compared to untreated PbA-infected mice.

### Transcriptional profiling confirms a systemic effect of oAC treatment

**Figure 3:**
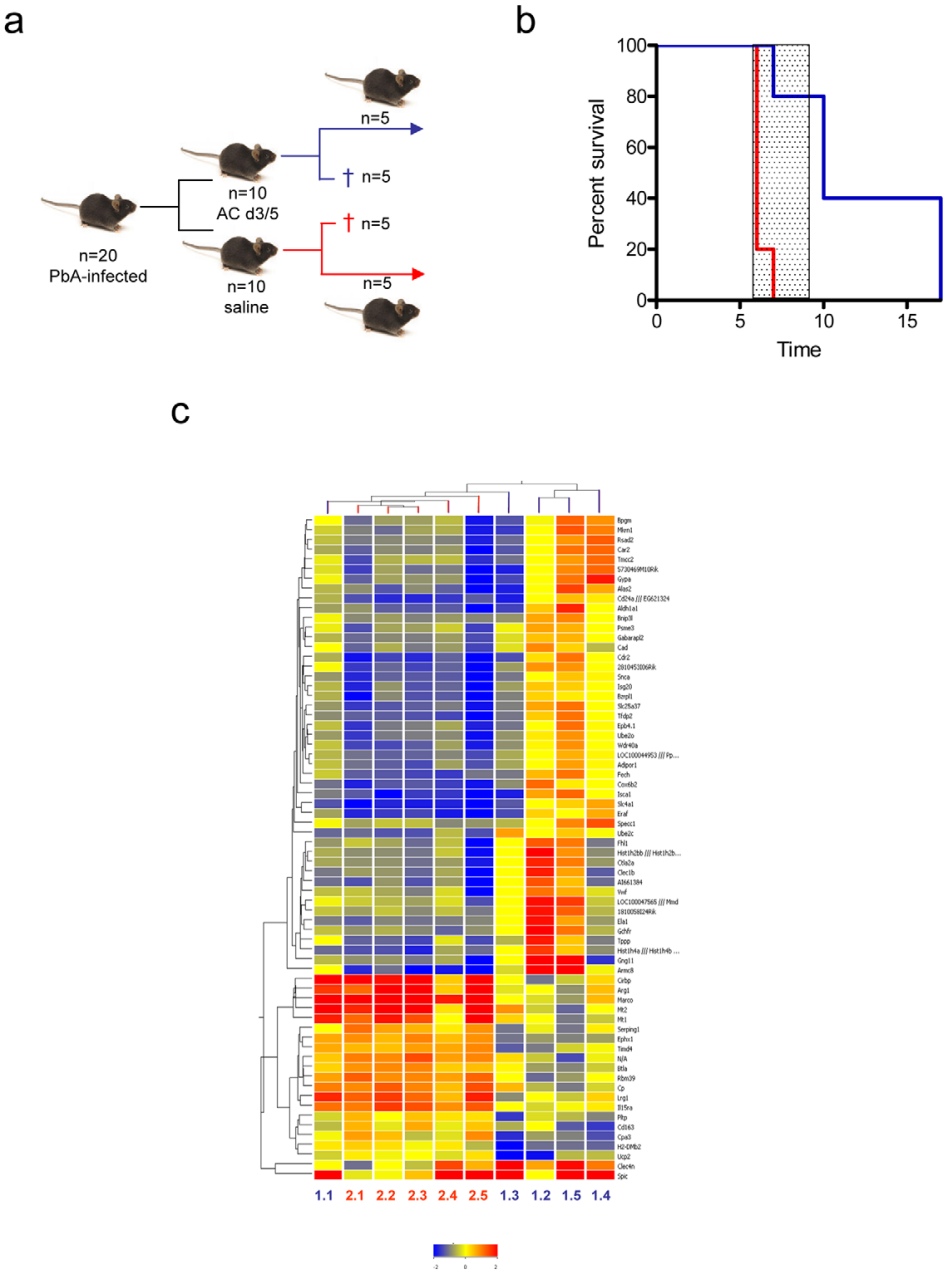
Transcriptional profiling of oAC-treated and untreated PbA-infected mice. **a**) 20 C57BL/6 mice were infected with PbA and at d3 and d5, 10 were treated with oAC. At day 6, 5 mice per group were killed for gene expression analysis and the remainder monitored for survival. **b**) oAC-treated PbA-infected mice (blue line) were significantly protected from ECM compared to untreated PbA-infected mice (red line; p = 0.048). **c**) Heat map generated by hierarchical clustering of the 68 genes that passed the p-value threshold of a false discovery rate of 5% and were >2-fold differentially expressed in whole blood of oAC-treated vs. untreated PbA-infected mice. Gene tree (side) and sample tree (top) are shown. Individual mice are numbered at bottom (oAC treated: 1.1–1.5; blue text, Untreated: 2.1–2.5; red text). Heat map intensity scale is also shown.

In order to determine whether oAC had further measurable systemic effects, we performed global gene expression profiling on whole blood. The experimental design is summarised in **Fig. 3a**. 20 mice were infected with PbA and at d3 and d5, 10 were treated with oAC (Group 1) and 10 with saline (Group 2). At d6 p.i., after the onset of ECM symptoms but before any deaths had occurred in Group 2, 5 mice from each group were killed, and whole blood processed for gene chip analysis and flow cytometry. 5 naïve mice provided blood as reference (‘baseline’). The remaining infected mice were used to monitor the development of ECM, again showing that oAC significantly protected mice from ECM (Fisher's Exact test p = 0.048; RR =  0.2; **Fig. 3b**). At the time of sampling for gene expression analysis, mice in both groups had similar parasitemias, mean body weight, and frequencies of T cells (CD3^+^), B cells (B220^+^) and NK cells (NK1.1^+^), whereas CD11b^+^ cells were slightly reduced in frequency in the blood of mice treated with oAC (data not shown). We identified a whole blood signature associated with oAC treatment of PbA-infected mice, comprised of 68 genes with a false discovery rate corrected P value of <0.05 (ranging from p = 0.011 to p = 1.47e^7^) and which passed a cut-off of 2-fold up- or down-regulation, relative to untreated PbA-infected mice (**Fig. 3c**). This representation shows that there was a high degree of similarity in the whole blood expression profiles of untreated PbA-infected mice. However, within the oAC-treated group, there was more variability, with some mice (most notably #1.1 and to a lesser extent #1.3) having a gene expression profile that more closely resembled that of untreated mice. Given the level of protection against ECM observed in the remainder of this cohort of oAC-treated mice (**Fig 3b**), this analysis suggests that whole blood profiling may be able to identify treatment failures, though further studies are required to substantiate this claim. Nevertheless, collectively our microarray and cytokine analysis confirmed that oAC treatment had a significant and systemic impact on the immune and inflammatory response in mice infected with PbA.

### An open labelled Phase I trial to evaluate the pharmacokinetics of AS in combination with oAC

The protective efficacy and immunomodulatory effects of oAC, as demonstrated above, supported a potential role for oAC in the treatment of human malaria. Exploration of oAC as adjuvant treatment for severe malaria has to take into account, however, that oAC increases the elimination of quinine [24]–[26], up to now the mainstay of treatment for severe malaria (SM). Recognizing that quinine is likely to be replaced by AS for the treatment of severe malaria [14], as an important first safety step, we therefore sought to determine whether oAC altered the pharmacokinetics of parenteral AS or its active metabolite DHA.

**Figure 4:**
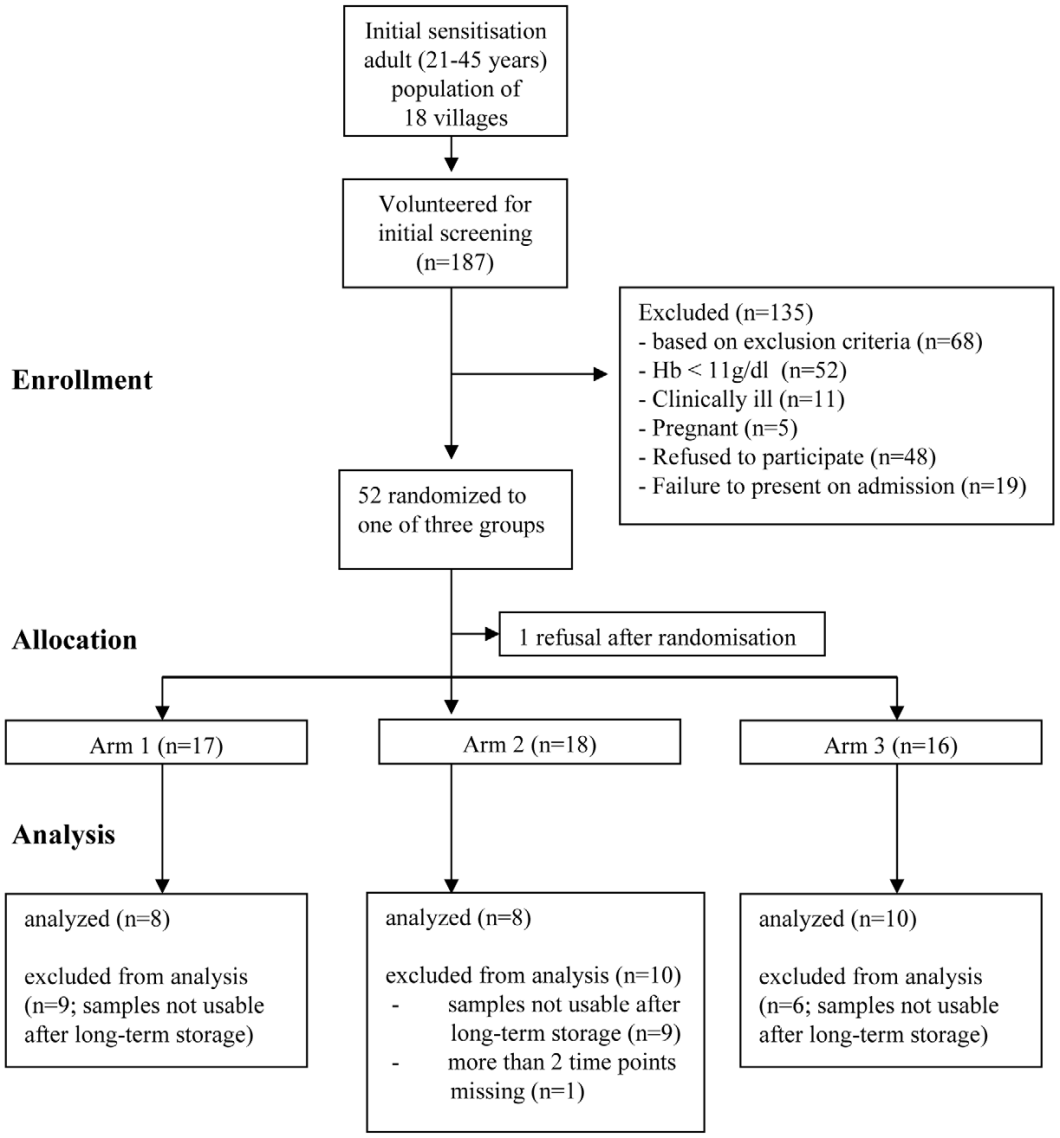
Study flow chart. The chart provides information on the number of individuals for each stage. The figures in brackets indicate the number of samples that could be analysed for AS and DHA.

Fifty-two healthy adults were enrolled in the study (from 187 screened volunteers (**Fig. 4**). The study participants in each group were similar with regard to age, weight, administered AS dose and gender distribution (**Table S1)**. The study drugs were safe and well tolerated. No serious adverse event occurred. One participant in arm 1 (AS alone) vomited once after administration of the i.v. AS. No clinically relevant deviations from the normal ranges were observed for the biochemistry and FBC tests.

**Figure 5:**
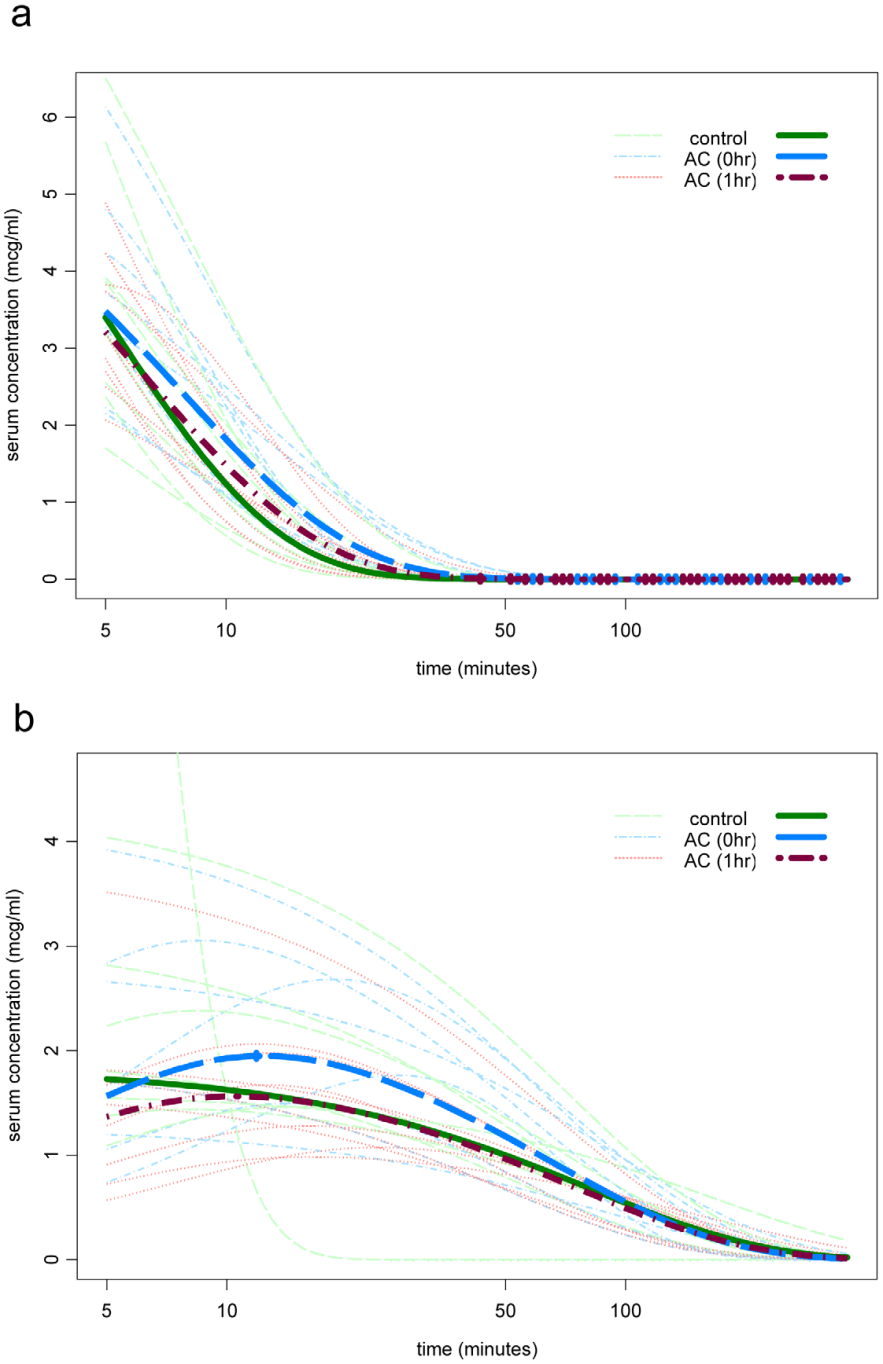
Pharmacokinetics of AS with and without co-administration of oAC. **a**) AS and **b**) DHA plasma concentrations in normal volunteers treated with AS alone (green), treated with AS and oAC simultaneously (blue) or with AS and 1 h later with oAC (red). Data are shown as fitted curves using a one-compartment model for each individual (n = 8 for arm 1 and 2 and n = 10 for arm 3) and also as a curve fitted through the geometric mean concentration for each arm at each time point (heavy lines). The geometric mean AUC was not statistically significantly different between the three arms (p = 0.92 for AS, and p = 0.55 for DHA by ANOVA).

AS and DHA plasma concentrations were assessed for 8 individuals in each of arms 1 and 2, and 10 individuals in arm 3 as described. **Fig. 5** shows each fitted curve, and a one-compartment model fitted through the geometric mean concentration for each arm at each time point, using the geometric mean dose for that arm. For both AS (**Fig. 5a**) and DHA (**Fig. 5b**), the geometric mean AUC was similar between the three arms. ANOVA analysis comparing the area under the curve (AUC, calculated as dose/clearance) shows no significant difference between the three groups (p = 0.92 for AS, and p = 0.55 for DHA). Compared to controls, the largest difference of the four comparisons in geometric means was −17%, which occurred for DHA in arm 3. None of the comparisons versus control of any of the pharmacokinetic parameters reached statistical significance. For AUC, the lowest of the four lower confidence limits was −43% versus control (DHA; arm 3) and the highest of the upper limits was +42% (AS; arm 2) (**Table 1**). Hence we were not able to detect any effect of oAC on the pharmacokinetics of AS or DHA. Even though the sample size was small, we were able to establish that any effect on AUC was unlikely to be as much as 50%, a slight effect compared to the ∼200% variation observed between individuals within each arm.

**Table 1:** Pharmacokinetic parameters for artesunate and DHA in the three trial arms.

drug and parameter	AS + water (control) (*n* = 8)	AS + charcoal simultaneously (*n* = 8)		AS + charcoal 1 hour later (n = 10)	
			percent difference from control (95% CI)		percent difference from control (95% CI)
Artesunate					
AUC (min×*µ*g/ml)	48 (23–77)	49 (32–71)	+2 (−26, +42)	46 (33–67)	−4 (−29, +32)
t_1/2_ (min)	3.4 (1.8–5.2)	4.1 (2.9–5.3)	+21 (−11, +65)	4.0 (2.6–7.0)	+18 (−12, +58)
CL/F (liters/min)	3.1 (1.5–5.5)	3.1 (2.2–4.5)	+2 (−27, +44)	3.1 (2.0–4.3)	+1 (−27, +40)
V/F (liters)	15 (7.6–29)	19 (9.2–34)	+24 (−22, +98)	18 (11–34)	+19 (−23, +85)
DHA					
AUC (min×*µ*g/ml)	172 (108–310)	161 (91–268)	−6 (−37, +38)	142 (91–247)	−17 (−43, +19)
t_1/2_ (min)	34 (1–116)	40 (19–65)	+20 (−49, +183)	52 (32–109)	+53 (−32, +245)
CL/F (liters/min)	0.85 (0.46–1.27)	0.95 (0.57–1.56)	+12 (−24, +64)	1.00 (0.53–1.73)	+16 (−18, +70)
V/F (liters)	41 (2–113)	56 (30–132)	+34 (−43, +218)	75 (34–167)	+80 (−20, +309)
C_max_ (*µ*g/ml)	2.9 (1.4–29)	2.2 (1.3–4.1)	−25 (−61, +45)	1.7 (1.0–3.8)	−43 (−69, +7)
t_max_ (min)	0.8 (0–14)	0.7 (0–27)	−9 (−97, +2740)	2 (0–24)	+162 (−90, +6720)

## Discussion

Each year, infection with *Plasmodium falciparum* causes 400–600 million cases of malaria and up to 1 million childhood deaths in Africa [1] Here, we demonstrate that oAC protects mice from death due to ECM, with associated changes in T cell cytokine production and in whole blood gene expression profiles. Importantly, the changes in immune response and gene expression profile observed following oAC treatment did not impact negatively on host protection against PbA infection *per se*. In a randomized controlled open label Phase I study, we found no interference of oAC with the pharmacokinetics of the currently best available anti-malarial drug AS. Collectively, these data pave the way for clinical evaluation of oAC as a new safe, effective and affordable adjunct treatment for severe malaria in man.

Our data indicate that oAC almost completely inhibited the clinical and histopathological signs of ECM in mice. In those mice that did develop ECM, onset was delayed, and in surviving mice oAC also appeared to provide a degree of protection against death due to high parasitemia, as measured by overall survival time. However, further studies are clearly needed to understand the mechanism(s) by which oAC mediates these striking effects. In chronic kidney disease, it has been proposed that oAC serves as a sink to block intestinal absorption of indole, thereby limiting hepatic production of indole sulphate, a regulator of TGFβ production [13]. Other data suggest the possibility of bile-mediated transfer of cytokines to the intestinal lumen via the entero-hepatic pathway, with charcoal acting as a presumptive local adsorbant [12].

Two lines of evidence from the current study suggest that oAC has broad systemic effects on immune and inflammatory processes. First, oAC treatment significantly reduced the number of splenic CD4^+^ and CD8^+^ T cells capable of TNF^+^ production and of CD4^+^ T cells co-producing TNF and IFNγ, as determined after re-stimulation. Although no effect of oAC was observed on cytokine production measured directly ex vivo, such an observation is in keeping with reports that serum pro-inflammatory cytokines, included those measured above, peak earlier in infection than the time point used in our analysis [27] and with the data of others that indicates a greater ability to detect multi-functionality after re-stimulation [23]. Similar associations between disease outcome and cytokine levels have been observed in other models in which the course of ECM has been altered. For example, in MyD88^−/−^ C57BL/6 mice, which are resistant to ECM, production of IFNγ, TNF and IL-17 was reduced [27]. Similarly, in mice treated other newly proposed adjunct therapies that show similar levels of efficacy against ECM as demonstrated here for oAC, including panthenine [28] and rosiglitazone [29], levels of TNF were also reduced. For rosiglitazone, dampening of inflammation and enhanced parasite clearance have also been observed in humans given this drug as adjunct therapy (with atovaquone/proguanil) for mild malaria [30], confirming the translational potential of studies in murine models of disease.

Second, microarray analysis identified a clear whole blood transcriptional signature that distinguished oAC-treated from untreated PbA-infected mice. Gene expression analysis in ECM has previously been largely restricted to using spleen cells or brain tissue [31]–[33] making comparisons across either genetically disparate hosts or using different parasite strains. In contrast, in the application of this technology to human malaria has been restricted to analysis of PBMC or whole blood [34]–[36], again usually comparing individuals with distinct disease outcome. To our knowledge, no direct comparisons of gene expression profile have been made following interventions that seek to prevent the development of severe disease. Further analysis of our gene expression data, including refinement of the signature associated with oAC treatment, will be published elsewhere. The data shown here nevertheless provides evidence that oAC treatment directly or indirectly affected gene regulation as determined in whole blood from PbA-infected mice. The signature of 68 genes associated with oAC treatment included multiple genes involved in the acute phase response and inflammation, as well as genes involved in heme biosynthesis and erythrocyte function (e.g. *Fech*, *Epb4.1*, *and Slc25a37*). A case for the development of blood transcriptional biomarkers has been extensively argued elsewhere [37], [38], and the application of a ‘modular’ approach to genomic analysis of the response of SLE patients to treatment has been described recently [39]. It will be important in the future to determine whether similar or distinct signatures are associated with other interventions designed to interrupt the progression of ECM, and when oAC is evaluated for protection against CM and severe disease in man, to likewise determine whether such signatures have cross-species predictive value.

Although a molecular mode of action for oAC has yet to be identified, we believe that the experimental data reported here should nevertheless now provide a basis for the evaluation of oAC as a treatment for severe malaria. oAC may provide a first line therapy in the immediate absence of alternate treatment. Severe malaria is an acute illness that may present with neurological symptoms occurring often within 96 h of the onset of fever; much of this time may be spent traveling from remote villages to health clinics and consequently many children arrive in coma. If our observations from the murine model would translate to man, oAC given early in the course of infection could prevent the development of CM and may reduce CM-associated mortality. It is notable that AC has been used for many years in the treatment of poisoning, and can be given orally or via a naso-gastric tube, particularly in powdered form. It is well tolerated, has an excellent, well-documented safety profile, and is relatively inexpensive. Furthermore, AC has a long shelf life and can be stored at ambient temperatures. Should clinical efficacy be proven, these attributes would make AC highly suited for use in remote rural communities where it could be administered at the first point of care, for instance by a village health worker, and would encourage the effective uptake of charcoal therapy in malaria-endemic countries.

Nevertheless, given that specific treatment for malaria is available, and that oAC treatment alone had no anti-malarial activity in mice, the straightforward approach of assessing the efficacy of oral AC as a stand-alone therapy would be unethical in humans. Indeed, further exploration of the potential benefits of oAC as adjuvant treatment have to take into account that oAC is officially recommended to treat intoxication with quinine [40], increasing the elimination of this drug [24]–[26]. However, it was unknown whether the pharmacokinetics of AS, a drug now regarded to be superior to quinine for the treatment of severe malaria [14] and known to have a high endogenous clearance rate [41], would be affected by co-administration of oAC. We found no evidence of reduced plasma levels of AS or DHA due to oAC, when given simultaneously with AS, or when administered 1 h later. In arms 2 (AS and oAC simultaneously) and 3 (oAC 1 h after AS), individuals received their first dose of oAC 12 h prior to starting plasma sampling. The fact that the AS and DHA concentrations found in these groups were not lower than those observed in the control group support the conclusion that even a high concentration of AC in the intestine at the time of administration of AS did not interfere with the pharmacokinetics of AS or DHA. Although the trial did not have an equivalence design, all subjects who started therapy followed the protocol schedule and there was no rescue therapy. This reduces concerns about interpreting the results as if they had come from an equivalence trial. Although the sample size required for an equivalence trial would have been different, the sample size actually achieved (which was reduced by the failure to analyse some samples) is of course reflected in the width of the confidence intervals. These data therefore suggest that clinical trials of combined AS and oAC treatment in patients with malaria could proceed without compromising the anti-malarial activity of AS.

Finally, with the limited resources available at the primary health care level, it is often not possible to reliably assign the correct diagnosis to a severely ill child, especially to differentiate between malaria and a bacterial infection (e.g. pneumonia, typhoid/non-typhoid salmonella or meningitis) [42], [43]. Treatment is mainly empirical on a presumptive diagnosis, and clinical assessment is often disturbingly poor [44] resulting in an over-diagnosis of malaria at the expense of severe bacterial infections [45], [46]. For an adjuvant treatment to be employed successfully, it needs to be safe and, ideally, beneficial irrespective of the precise diagnosis. oAC could be the ideal candidate for this purpose due to its non-specific anti-inflammatory potential, exploited successfully for over a decade to prevent progression of chronic kidney disease [13].

In conclusion, oAC prevented mortality associated with ECM in a mouse model and when given to humans did not interfere with the pharmacokinetics of AS or DHA. oAC therefore has the potential to be a readily-implemented therapy for the treatment of severe malaria. We suggest there is now an urgent need for controlled clinical trials to evaluate the efficacy of oAC in human malaria. We propose that the next step in the development of oAC should be to evaluate its safety profile as an adjuvant therapy in cases of uncomplicated malaria, a path recently taken for the development of rosiglitazone [30], before progressing to an evaluation of its potential benefit in cases of severe malaria. Incorporation of gene expression profiling into such trials should also yield further insights into the mode of action of AC in man.

## Supporting Information

Checklist S1 CONSORT Checklist S1
(0.20 MB DOC)

Protocol S1 Trial Protocol
(0.19 MB PDF)

Table S1 Characteristics of the study population according to study group.
(0.03 MB DOC)
