# Supplementary material for: Oral Activated Charcoal Prevents Experimental Cerebral Malaria in Mice and in a Randomized Controlled Clinical Trial in Man Did Not Interfere with the Pharmacokinetics of Parenteral Artesunate
Source: PLoS One. 2010 Apr 15;5(4):e9867. doi: 10.1371/journal.pone.0009867 (PMC2855344; doi:10.1371/journal.pone.0009867)
Supplement: Checklist S1 — CONSORT Checklist S1 (0.20 MB DOC) [file pone.0009867.s001.doc]

#
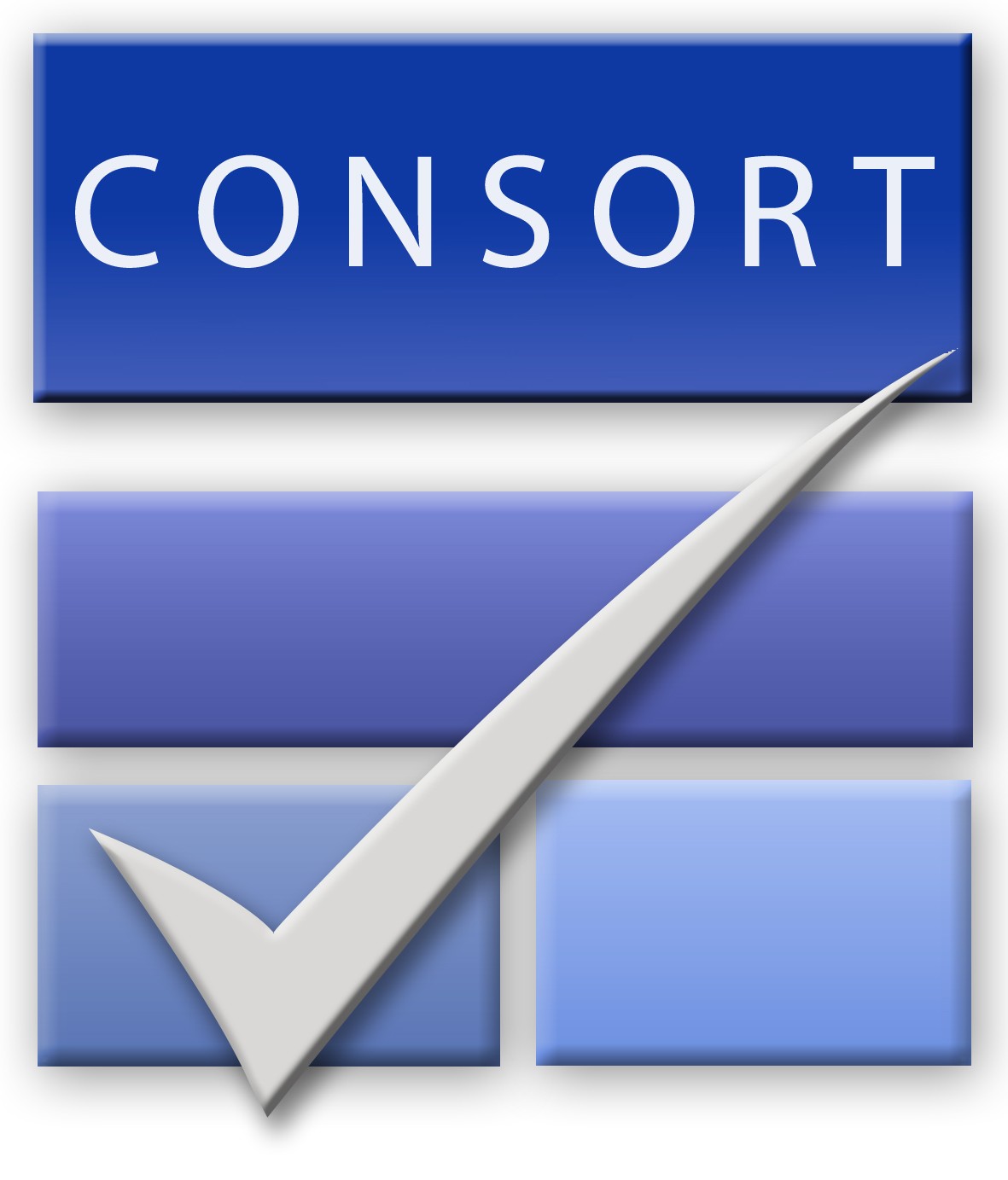
CONSORT Statement 2001 Checklist

**Items to include when reporting a randomized trial**

| ***PAPER SECTION* And topic** | Item | **Descriptor** | **Reported on**  **Page #** |
| --- | --- | --- | --- |
| TITLE & ABSTRACT | 1 | [How participants were allocated to interventions](http://www.consort-statement.org/index.aspx?o=1107) (*e.g*., "random allocation", "randomized", or "randomly assigned").  The abstract states: “a randomized controlled open label trial in 52 human volunteers” (ISRCTN NR. 64793756)” | Methods / Principal Findings |
| *INTRODUCTION* Background | 2 | [Scientific background and explanation of rationale](http://www.consort-statement.org/index.aspx?o=1016). The rationale to evaluate oral activated charcoal is described in the introduction. “Three observations led us to examine a potential role for activated charcoal (AC) in the treatment of severe malaria. First, a number of studies have demonstrated that in *ex vivo* haemofiltration, AC is highly effective at adsorbing a range of endotoxin-induced cytokines from the bloodstream, including TNF, IL-1 and IL-6 [11]. Second, TNF-dependent lethality in models of endotoxemia is associated with delivery of TNF to the intestinal lumen via the bile duct. Thus, bile duct cannulation protects rats from lethal endotoxemia [12] (Ulloa et.al. unpublished), raising the possibility that AC in the intestinal lumen might directly affect cytokine availability. Third, oAC has for many years been used in the clinic to suppress chronic kidney disease, through indirect modulation of inflammation”  Further, the results from the animal studies presented in this paper do constitute a strong rationale to explore oral activated charcoal in humans | Introduction, second § |
| *METHODS* Participants | 3 | [Eligibility criteria for participants](http://www.consort-statement.org/index.aspx?o=1017#3a) and the [settings and locations where the data were collected](http://www.consort-statement.org/index.aspx?o=1017#3b). | Under: Open label phase 1 trial: Study population and design |
| Interventions | 4 | [Precise details of the interventions intended for each group and how and when they were actually administered](http://www.consort-statement.org/index.aspx?o=1021). | Under: Open label phase 1 trial: Study population and design |
| Objectives | 5 | [Specific objectives and hypotheses](http://www.consort-statement.org/index.aspx?o=1022). | Under: Open label phase 1 trial: Study population and design |
| Outcomes | 6 | [Clearly defined primary and secondary outcome measures](http://www.consort-statement.org/index.aspx?o=1023#6a) and, when applicable, any [methods used to enhance the quality of measurements](http://www.consort-statement.org/index.aspx?o=1023#6b) (*e.g.*, multiple observations, training of assessors). | Under: measurement of AS and DHA |
| Sample size | 7 | [How sample size was determined](http://www.consort-statement.org/index.aspx?o=1024#7a) and, when applicable, [explanation of any interim analyses and stopping rules](http://www.consort-statement.org/index.aspx?o=1024#7b). | Under: data handling and Statistical analysis – clinical data |
| Randomization -- Sequence generation | 8 | [Method used to generate the random allocation sequence, including details of any restrictions](http://www.consort-statement.org/index.aspx?o=1025) (*e.g*., blocking, stratification) | Under: Open label phase 1 trial: Study population and design |
| Randomization -- Allocation concealment | 9 | [Method used to implement the random allocation sequence](http://www.consort-statement.org/index.aspx?o=1026) (*e.g*., numbered containers or central telephone), clarifying whether the sequence was concealed until interventions were assigned. | Under: Open label phase 1 trial: Study population and design |
| Randomization -- Implementation | 10 | [Who generated the allocation sequence, who enrolled participants, and who assigned participants to their groups](http://www.consort-statement.org/index.aspx?o=1027). | Under: Open label phase 1 trial: Study population and design |
| Blinding (masking) | 11 | [Whether or not participants, those administering the interventions, and those assessing the outcomes were blinded to group assignment](http://www.consort-statement.org/index.aspx?o=1028#11a). If done, [how the success of blinding was evaluated](http://www.consort-statement.org/index.aspx?o=1028#11b). The investigator administering the study drugs was not blinded to the nature of the intervention; as stated several times, this was an open label trial. However, the investigators measuring the pharmacokinetic parameters or the biochemistry or FBCs all dealt with samples, labeled with a 3-digit identifier that does not allow disclosure of the intervention. | Under: Open label phase 1 trial: Study population and design and: measurement of AS and DHA |
| Statistical methods | 12 | [Statistical methods used to compare groups for primary outcome(s)](http://www.consort-statement.org/index.aspx?o=1029#12a); [Methods for additional analyses](http://www.consort-statement.org/index.aspx?o=1029#12b), such as subgroup analyses and adjusted analyses. | Data handling and statistical analysis-clinical data |
| RESULTS Participant flow | 13 | [Flow of participants through each stage](http://www.consort-statement.org/index.aspx?o=1018) (a diagram is strongly recommended). Specifically, for each group report the numbers of participants randomly assigned, receiving intended treatment, completing the study protocol, and analyzed for the primary outcome. [Describe protocol deviations from study as planned, together with reasons](http://www.consort-statement.org/index.aspx?o=1086). | Please see figure 4 and Data handling and statistical analysis-clinical data |
| Recruitment | 14 | [Dates defining the periods of recruitment and follow-up](http://www.consort-statement.org/index.aspx?o=1087). | Open label phase 1 trial study population and design |
| Baseline data | 15 | [Baseline demographic and clinical characteristics of each group](http://www.consort-statement.org/index.aspx?o=1088). | An open labelled Phase I trial to evaluate the pharmacokinetics of AS in combination with oAC.  and suppl. Table 1 |
| Numbers analyzed | 16 | [Number of participants (denominator) in each group included in each analysis and whether the analysis was by "intention-to-treat"](http://www.consort-statement.org/index.aspx?o=1089). State the results in absolute numbers when feasible (*e.g*., 10/20, not 50%). | An open labelled Phase I trial to evaluate the pharmacokinetics of AS in combination with oAC. |
| Outcomes and estimation | 17 | [For each primary and secondary outcome, a summary of results for each group, and the estimated effect size and its precision](http://www.consort-statement.org/index.aspx?o=1090) (*e.g.*, 95% confidence interval). | See table 1 |
| Ancillary analyses | 18 | [Address multiplicity by reporting any other analyses performed](http://www.consort-statement.org/index.aspx?o=1091), including subgroup analyses and adjusted analyses, indicating those pre-specified and those exploratory. | Not applicable |
| Adverse events | 19 | [All important adverse events or side effects in each intervention group](http://www.consort-statement.org/index.aspx?o=1092). | None occurred see: An open labelled Phase I trial to evaluate the pharmacokinetics of AS in combination with oAC. |
| *DISCUSSION* Interpretation | 20 | [Interpretation of the results](http://www.consort-statement.org/index.aspx?o=1019), taking into account study hypotheses, sources of potential bias or imprecision and the dangers associated with multiplicity of analyses and outcomes. | Discussion |
| Generalizability | 21 | [Generalizability (external validity) of the trial findings](http://www.consort-statement.org/index.aspx?o=1094). | Discussion, |
| Overall evidence | 22 | [General interpretation of the results in the context of current evidence](http://www.consort-statement.org/index.aspx?o=1095). | Discussion |

*From* Moher D, Schulz KF, Altman DG. The CONSORT statement: revised recommendations for improving the quality of reports of parallel-group randomised trials. Lancet 2001; 357(9263):1191-1194.

**The CONSORT Statement 2001 checklist is intended to be accompanied with the explanatory document that facilitates its use. For more information, visit** [**www.consort-statement.org**](http://www.consort-statement.org/)**.**
