# Supplementary material for: Oral Activated Charcoal Prevents Experimental Cerebral Malaria in Mice and in a Randomized Controlled Clinical Trial in Man Did Not Interfere with the Pharmacokinetics of Parenteral Artesunate
Source: PLoS One. 2010 Apr 15;5(4):e9867. doi: 10.1371/journal.pone.0009867 (PMC2855344; doi:10.1371/journal.pone.0009867)
Supplement: Protocol S1 — Trial Protocol (0.19 MB PDF) [file pone.0009867.s002.pdf]

The Gambia

CLINICAL TRIAL PROTOCOL

SCC Number 1025

Evaluating the potential role of oral activated charcoal as an adjunct treatment for severe bacterial infections and severe malaria

---

Short Title:	Charcoal
ISRCTN:	64793756
Protocol Version:	6.0 – 21 November 2006

Sponsor:
Medical Research Council Laboratories, Fajara,
PO Box 273, Banjul,
The Gambia, West Africa

Principal investigator:
Dr Michael Walther,
Malaria Research Programme
MRC Laboratories, Fajara,
PO Box 273, Banjul
The Gambia, West Africa

Author:
Dr Uduak Okomo,
MRC Laboratories, Fajara, The Gambia

Confidential & Proprietary Information
The present document is to be used by MRC representatives, investigators, regulatory authorities, institutional review boards and ethics committees. This document is confidential and cannot be communicated to third parties or used for publication or conferences without written approval of the sponsor (MRC Laboratories, The Gambia)

SIGNATURE PAGE

**Principal Investigator**:<br>Dr Michael Walther<br>Malaria Research Programme<br>MRC Laboratories, Fajara<br>PO Box 273 Banjul<br>The Gambia<br>Phone: (+220) 4497928<br>Email: mwalther@mrc.gm	**Date:**	**Signature:**

**Statistician:**<br>Dr Neal Alexander<br>London School of Hygiene and Tropical Medicine<br>London<br>United Kingdom<br>Phone:+5728924221<br>Email: Neal.Alexander@lshtm.ac.uk	**Date:**	**Signature:**

**Safety Monitor:**<br>Dr Samuel Dunyo<br>Head of Station,<br>MRC Laboratories, Farafenni Field Station<br>Email: sdunyo@mrc.gm	**Date:**	**Signature:**

INVESTIGATOR'S SIGNATURE PAGE

By my signature below I hereby confirm that I will conduct the trial described in the approved version of Protocol No 1025, in compliance with MRC Guidelines for Good Clinical Practice in Clinical Trials and the Good Clinical Practice: Consolidated Guideline by the International Conference on Harmonization (ICH GCP Principles) and in accordance to local legal and regulatory requirements.

**Investigator:**<br>Dr. Uduak Okomo	**Date:**	**Signature:**
**Institution:**<br>MRC Laboratories, Fajara, PO Box 273, Banjul, The Gambia<br>Phone: (+220) 4497114		

Table of Contents

Page
Signature Page ii
Investigator's Signature Page iii
List of Abbreviations 6
Protocol Summary 7
1 Key Roles 9
2 Background Information and Rationale 11
2.1 Background Information 11
2.2 Rationale 12
2.3 Potential Risks and Benefits 13
3 Trial Objectives 15
3.1 Trial Objectives 15
3.2 Trial Endpoints 15
4 Trial Design 16
4.1 Type of Trial and Design 16
4.2 Randomisation Procedure 16
4.3 Investigational Product 16
4.3.1 Artesunate 16
4.3.2 Activated Charcoal 17
5 Selection and Withdrawal of subjects 18
5.1 Selection of Subjects 18
5.2 Eligibility of Subjects 18
5.3 Withdrawal of Subjects 18
6 treatment of subjects 20
7 Trial Procedures and Evaluations 21
7.1 Trial Schedule 21
7.1.1 Screening 21
7.1.2 Enrolment 21
7.1.3 Follow-up 22
7.2 Trial Evaluations 22
7.2.1 *Clinical Evaluations* 22
7.2.2 *Laboratory Evaluations* 22
8 Safety considerations 23
9 Statistical Considerations 24
10 Direct Access to Source Data 25
11 Quality Control and Quality Assurance 25
12 Ethics 26
12.1 Informed Consent Process 26
12.2 Subject Confidentiality 26
12.3 Biohazard Containment 27

13 Data Handling and Record Keeping..28
14 INDEMNITY 30
15 PLANS FOR DISTRIBUTION OF RESEARCH FINDINGS 30
16 Literature References...30
Appendix A: Summary Participant Schedule 33

LIST OF ABBREVIATIONS

AC	Activated Charcoal
AE	Adverse Event/Adverse Experience
AR	Adverse Reaction
ARS	Artesunate
AUC	Area under the concentration-time curve
Cl	Clearance
CRF	Case Report Form
CSF	Cerebrospinal Fluid
DHA	Dihydroartemisinin
FBC	Full Blood Count
FW	Field Worker
GCP	Good Clinical Practice
IB	Investigator’s Brochure
ICH	International Conference on Harmonization
ITN	Insecticide Treated Net
EC	Ethics Committee
ISRCTN	International Standard Randomised Controlled Trial Number
LI	Lead Investigator
LPS	Lipopolysaccharide
MRC	Medical Research Council
N	Number (typically refers to subjects)
PABA	Para-aminobenzoic Acid
PI	Principal Investigator
PCR	Polymerase Chain Reaction
SAE	Serious Adverse Event
SAR	Serious Adverse Reaction
SCC	Scientific Coordinating Committee
SEN	State Enrolled Nurse
SOP	Standard Operating Procedure
SRN	State registered Nurse
TMF	Trial (site) Master File (Regulatory File)
TSC	Trial Steering Committee
Vd	Volume of Distribution

PROTOCOL SUMMARY

**Title:**	Evaluating the potential role of oral activated charcoal as an adjunct treatment for severe bacterial infections and severe malaria – Preliminary pharmacokinetic study
**Short title**	Charcoal
**Phase:**	1
**Population:**	Healthy Gambians aged 21-45 years living in Farafenni area, The Gambia.
**Number of Sites:**	One site, the MRC ward in the AFPRC hospital in Farafenni, The Gambia.
**Study Duration:**	5 months
**Subject Participation Duration:**	18-24 hours
**Description of Products:**	1. Intravenous Artesunate 2.4mg/kg bodyweight 12 hourly x 2 doses<br>2. Oral activated charcoal 50mg 12 hourly x 2 doses
**Objectives:**	To evaluate whether orally given AC alters the pharmacokinetic of intravenously applied artesunate when given simultaneously or after 1 hour.
**Description of Study Design:**	An open labeled randomized controlled pharmacokinetic study with 3 groups of 30 subjects each (total of 90 subjects). Artesunate will be given to all study participants intravenously, at a dose of 2.4mg/kg on day 0, and after 12 hours. AC (50g dissolved in 350mls of water) will be given orally to subjects in groups 2 and 3 simultaneously with, or 1 hour after the dose of intravenous artesunate respectively. Subjects in group 1 (Control) will receive i.v. artesunate only with 350mls of plain water.

Schematic of Study Design:

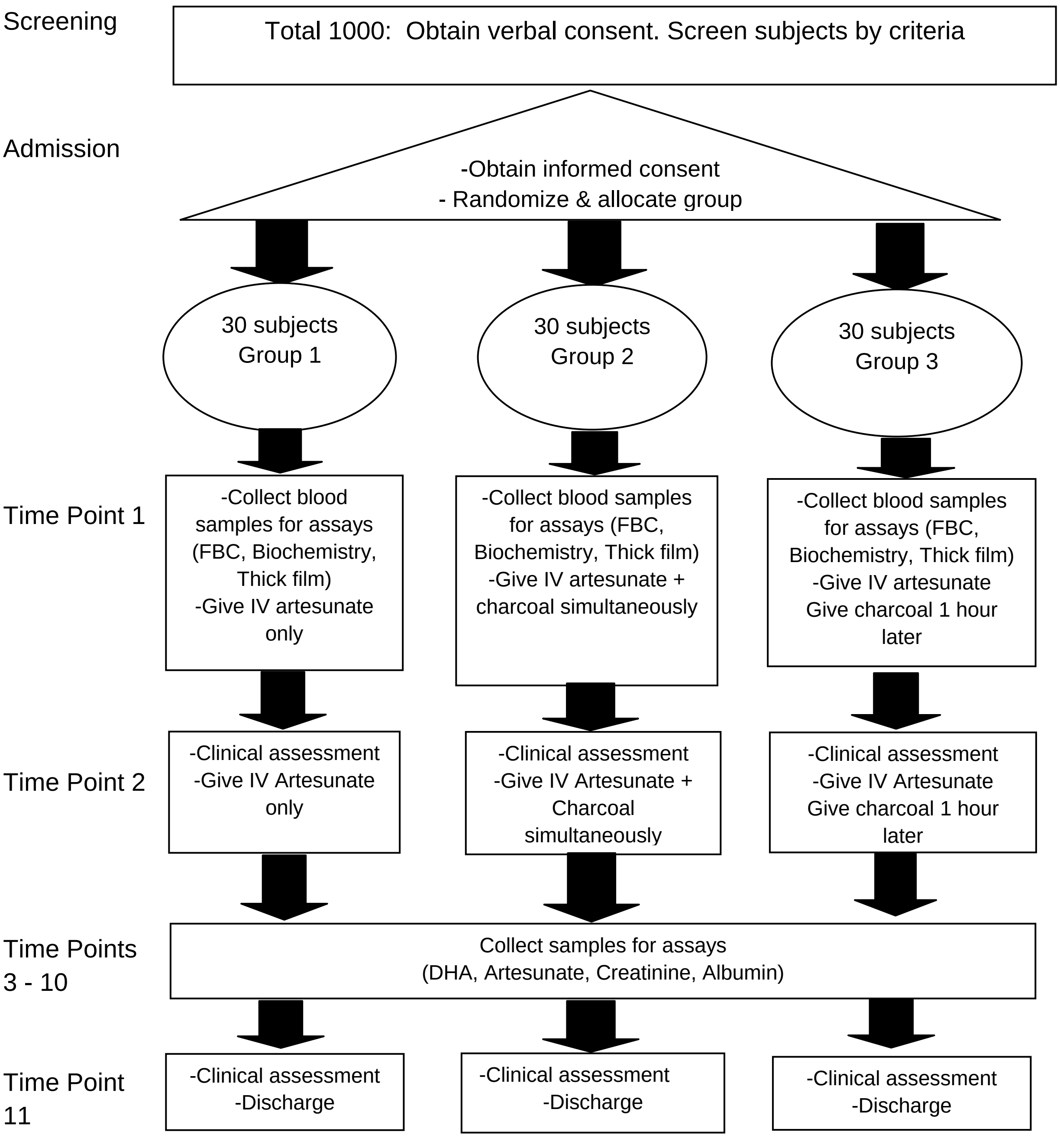

1 KEY ROLES

For questions regarding this protocol, contact: Dr. Michael Walther, Malaria Research Programme, MRC Laboratories, Fajara, P.O.Box 273, The Gambia.

For other questions, contact: Dr. Uduak Okomo, Clinical Services, MRC Laboratories, Fajara, P.O.Box 273, The Gambia.

**Principal Investigator:**	Dr Michael Walther,<br>Malaria Research Programme<br>MRC Laboratories, Fajara<br>PO Box 273 Banjul<br>The Gambia<br>Phone: (+220) 4496782, (+220) 4495442/6 Ext: 493<br>Email: mwalther@mrc.gm
**Investigator(s)**	Dr Uduak Okomo *MBBCh, MWACP*<br>MRC Laboratories, Fajara<br>PO Box 273, Banjul<br>The Gambia<br>Phone: (+220) 4497114<br>Email: uokomo@mrc.gm
**Trial Physicians**	Dr Uduak Okomo *MBBCh, MWACP*<br>MRC Laboratories, Fajara<br>PO Box 273, Banjul<br>The Gambia<br>Phone: (+220) 4497114<br>Email: uokomo@mrc.gm
	Dr Sanie Sesay<br>MRC Laboratories, Farafenni Field station<br>PO Box 273, Banjul<br>The Gambia<br>Phone: (+220) 9812632<br>Email: ssesay@mrc.gm
**Trial monitor**	Dr Jenny Mueller<br>Senior Scientific Officer Clinical Trials<br>MRC Laboratories, Fajara<br>PO Box 273Banjul, The Gambia<br>Phone: +220 449 54 42 Ext. 505<br>Fax: +220 449 79 24<br>E-mail: jmueller@mrc.gm

Safety Monitor:
Dr Samuel Dunyo *MBBs, PhD*
Head of Station, MRC Laboratories, Farafenni Field Station
Phone: (+220) 5735421
Fax: (+220) 5735512
Email: sdunyo@mrc.gm

Statistician
Dr Neal Alexander
London School of Hygiene and Tropical Medicine
London
United Kingdom
Phone:+5728924221
Email: Neal.Alexander@lshtm.ac.uk

Dr David Jeffries
MRC Laboratories, Fajara
PO Box 273, Banjul
The Gambia
Email: djeffries@mrc.gm

External Adviser
David C Warhurst, emeritus Professor
London School of Hygiene and Tropical Medicine
London WC1E 7HT United Kingdom
Phone Number: 44 (0)20 7 927 2341
Fax Number: 44 (0)20 7 637 0248
E-mail: david.warhurst@lshtm.ac.uk

Laboratory Research
Dr Harparkash Kaur
Lecturer in Pharmacology
London School of Hygiene & Tropical Medicine
E-mail: Harparkash.Kaur@lshtm.ac.uk

Dr Natalia Gomez-Escobar
Molecular Biologist
MRC Laboratories, Fajara
PO Box 273, Banjul, The Gambia
Phone: (+220) 4496782, (+220) 449544/6 Ext: 364
E-mail: ngescobar@mrc.gm

Data Manager
Ismaela Abubakar
Database manager
MRC Laboratories, Fajara
PO Box 273Banjul, The Gambia
E-mail: iabubakar@mrc.gm

2 BACKGROUND INFORMATION AND RATIONALE

2.1 Background Information

In Africa, severe malaria or pneumonia, sepsis or neonatal pneumonia account for ca. 46% of the 4.4 million yearly deaths in under 5 year olds(1).

A yet unpublished report by Ulloa et al. describes that in mice oral administration of activated charcoal (AC) improves survival in LPS-induced endotoxemia, during sepsis and malaria.

Using an established model, lethal polymicrobial peritonitis was induced by caecal ligation and puncture. Survival increased from 30% in controls to 80% in AC treated mice.
Peak serum TNF-α levels during LPS-induced endotoxemia were significantly reduced, and translated into a significant, dose-dependent reduction in mortality. Increased survival of charcoal treated mice correlated significantly with reduced serum levels of the high mobility group B1 (HMGB1) protein. This late acting cytokine appears in serum 18-24 hours after the onset of peritonitis(2, 3) and its blockade protects against LPS-induced mortality(4, 5), suggesting that AC protects against endotoxemia by attenuating serum HMGB1 levels.
Mice treated with AC 3 and 5 days post i.v. inoculation with 10^4 *P.berghei* ANKA - infected red blood cells were highly resistant to the development of cerebral malaria with a day 7 survival rate of 95% compared to 20% in untreated control mice. Brain biopsies revealed that the histological changes seen in control mice with cerebral malaria were not observed in mice treated with oral AC.

AC is a licensed, inexpensive drug that can be given orally or via a naso-gastric tube at high doses as a safe therapy of oral poisoning without major side effects(6-8).

In vitro, the antimalarial quinine showed a dose dependent adsorption capacity with activated charcoal(9) and repeated administration of oral charcoal increased the rate of elimination of a therapeutic dose of quinine in healthy volunteers(10). After treatment with repeated oral charcoal (50g 4 hourly), plasma quinine concentrations fell rapidly with a mean half-life of 8.1 +/- 1.1 h (s.d.) compared with more than 24 h in a previous report in similarly poisoned patients(11). The use of multi doses, activated charcoal became officially recommended in cases of quinine intoxication(6, 7).
In general, enhanced clearance of a drug upon co-administration of charcoal can be expected if the drug has a small volume of distribution (Vd), low binding affinity for plasma proteins, and a low endogenous clearance (Cl) (12).
Apart from quinine, artesunate, a water soluble artemisinin derivative that has a low Vd (0.15l/kg), but a high clearance rate of 50ml/kg/min can be used to treat severe malaria.

In South East Asia a randomized controlled multi center trial revealed that parenteral artesunate is superior to i.v. quinine for the treatment of severe malaria, reducing the mortality from 22% (quinine) to 15% (artesunate) (13). The planned AQUAMAT trial will evaluate this approach in several African sites including the Gambia, where it is conducted by the MRC unit's malaria programme.

Artesunate undergoes rapid biotransformation in the liver that yields the metabolite dihydroartemisinin (DHA) to which the beneficial antimalarial effect of artesunate is ascribed. A literature search performed by Prof. Warhurst and Dr Kaur has revealed that calculated pharmacological parameters do vary between settings, age of the subject and severity of disease (14-16) To design the sampling strategy an estimate of the half-life and T_{max} of artesunate after i.v. administration has been constructed based on data derived from healthy volunteers (see table 1 below).

Table 1: Reported half life ($t_{½}$) and time to reach maximum concentration (t_{max}) for artesunate and its active metabolite after i.v. administration

DRUG	$t_{1/2\ [min]}$	Clearance $_{[L/hr/kg]}$
i.v. artesunate	3.1 (± 1.1)	2.8 (±0.4)
Dihydroartemisin*	47 (± 14.8)	1.3 (±0.6)

*Artesunate is readily hydrolyzed to dihydroartemisinin, probably by blood esterases and the hepatic cytochrome P450 3A4.

2.2 Rationale

Given that AC is a licensed, inexpensive drug that can be given orally or via a naso-gastric tube at high doses as a safe therapy of oral poisoning without major side effects(6, 7) (8) we aim to explore its potentially life-saving properties in clinical trials in The Gambia. We plan to perform "proof of principle" studies for the use of AC as an adjuvant treatment in conditions such us severe bacterial infections, SIRS/sepsis and severe malaria in African children. In a series of randomized placebo controlled prospective studies clinical outcome of these conditions will be compared for standard treatment with and w/o AC co-administration.

Up to date, interactions between artesunate and oral AC have not been assessed. Given the potential impact of AC on quinine levels and taking into account that parenteral artesunate appears to be superior to quinine and may become the standard treatment for severe malaria in the near future, we decided to investigate the impact of AC on the pharmacokinetics of parenteral artesunate first.

For the initial step, we suggest here to study the impact of adjuvant oral AC on artesunate in healthy volunteers aged 21- 45 years. Given that both artesunate and activated charcoal are licensed drugs

and have an excellent safety record, we believe it is ethical to perform these trials in patients of this age group. In order to obtain meaningful results with regard to the planned efficacy trial in severely ill children, we aim to administer artesunate intravenously. This initial study is to demonstrate if oral AC impacts on the pharmacokinetics of i.v. artesunate.

Due to the extremely short half life of i.v. artesunate, the minimal sample size required per group is 30, thus a total of 90 subjects need to be enrolled. Subjects will be randomised and enrolled into one of the following groups:

1) artesunate i.v. without adjuvant treatment (control group) 350ml water given orally with each i.v. dose
2) artesunate i.v. and oral charcoal (50g) given simultaneously with each dose of artesunate
3) artesunate i.v. followed by oral charcoal (50g) given 1 hour after each dose of artesunate

2.3 Potential Risks and Benefits

The suggested doses of activated charcoal have been derived from official guidelines for treatment of poisoning that recommend 50-100g AC for adults, or 10-25g in children less than 5 years old, respectively. Activated Charcoal will be diluted in 350mls of water.
Oral activated charcoal is an inert, non-toxic drug. It may taste bitter and at higher doses may cause some constipation.

Artesunate will be given intravenously, at a dose of 2.4mg/kg on day 0, and after 12 hours. Mild tenderness, bruising, or fainting may result from venepuncture.
Intravenous artesunate is highly effective in killing malaria parasites and at the suggested dose has an excellent safety profile(13). However, it should be mentioned that in animals intramuscular administration of the oil based artemisinin derivatives artemether or arteether have produced an unusual selective pattern of damage to certain brain stem nuclei, particularly those involved in the auditory relay(17, 18). However, there is no evidence of similar damage in humans. Recent studies of auditory-evoked potentials in Vietnamese and Thai patients treated with artemisinin derivatives have provided reassuringly normal data(19). Moreover, in animal models the water-soluble compounds such as artesunate have given significantly less neurotoxicity than intra-muscular administration of the oil based compounds(20), and in a large cohort treated with either artesunate or artemether there was no evidence of any adverse nervous system effect(21). A recent study further demonstrated that no DHA was detectable in the CSF upon intravenous application of artesunate(22), and an up to date expert review concludes that artemisinin derivatives have no identifiable dose-related adverse effects in humans and only very rarely produce allergic reactions

All 90 study participants will be given an ITN at the end of the study. For a period of 2 months the study participants and their immediate family (wive(s), husband, biological children) will be granted

access to the MRC ward at the hospital. A SEN will accompany the screening team and will be equipped to treat minor acute ailments

Malaria remains an important cause of mortality in children under 5 years of age in Sub Saharan Africa including the Gambia. Studies in mice revealed that oral activated charcoal may have the potential to reduce the mortality from severe malaria substantially. Given that oral activated charcoal is an exceptionally safe and cheap drug, blessed with an endless shelf life, not having any expensive or sophisticated demands on storage or transport, this intervention would have the potential to be implemented on a broader scale fairly easy, if shown to be beneficial.
Results from this study would provide the first data on safety of co-administration of oral activated charcoal and intravenous artesunate, which is a pre-requisite for subsequent efficacy trials.

The study will also benefit the AFPRC hospital. A centrifuge, a water dispenser and a shelving system will be bought to improve the clinical trials facilities at the hospital, and salaries for 1 SEN, 1 SRN and 2 nurse attendees will be provided. Following the current agreement between the AFPRC hospital and the MRC, fuel for generators will be provided.

3 TRIAL OBJECTIVES

3.1 Trial Objectives

The primary objective is to evaluate whether orally given AC alters the pharmacokinetic of intravenously applied artesunate and its metabolite dihydroartemisinin (DHA) when given simultaneously or after 1 hour.

The general purpose is to study the pharmacokinetic interactions between artesunate and AC as part of a "proof of principle" studies for the use of AC as an adjuvant treatment in mild and severe malaria in African children.

3.2 Trial Endpoints

The plasma levels of artesunate and its metabolite DHA as well as para-aminobenzoic acid (PABA) will be measured by liquid chromatography – mass spectrometry (LC-MS) at the time points 5, 10, 15, 30, 60, 90 min and 3 and 6 hours after the second dose of artesunate has been given (at 12 hours) to determine their C_{max}, t_{max}, $t_{½}$ and AUC.

Creatinine and albumine will be measured too. These parameters are necessary to calculate renal clearance and binding of the drug tp plasma proteins.

4 TRIAL DESIGN

4.1 Type of Trial and Design

This controlled randomised open labelled phase 1 pharmacokinetic study will be conducted at the MRC ward in the AFPRC hospital in Farafenni, The Gambia as single site. Samples will be analyzed at the MRC Unit, Farafenni and Fajara and at The London School of Hygiene and Tropical Medicine in the UK.

The 90 participants will be randomized into three parallel study groups:

- 30 subjects in Group 1 as control, who will receive artesunate and water
- 30 subjects in Group 2 who will receive AC and artesunate simultaneously
- 30 subjects in Group 3 who will receive AC 1 hour after artesunate.

The study is expected to last 5 months. Subject recruitment and enrolment will last for 3 months. Each subject will stay in hospital for dosing and sample collection for 18 to 24h. The subsequent 2 months will be used to provide health care for study participants.

4.2 Randomisation Procedure

90 random numbers will be generated in blocks of 15 (e.g. 1-15, 16-30) using the random numbers generation function from Excel, Microsoft. In order of appearance, at the random numbers generator, these numbers will be allocated to group 1, 2 or 3, respectively, so that after each set of 15 numbers an equal distribution to all three groups is guaranteed. Cards with the group numbers 1-3 will then be put into envelopes labelled with the numbers that have been randomized to each group. The envelopes will then be arranged in ascending order. The envelopes will be allocated one after the other to eligible subjects in the order they are enrolled in the study.

4.3 Investigational Product

4.3.1 Artesunate

Artesunate for intravenous injection is manufactured by Guilin Pharmaceutical CO., Ltd, China. It comes as Artenusic acid in vials of 60-mg. The pack also contains a 1ml ampoule of 5% sodium bicarbonate as solvent.
Artesunate will be given to all study participants intravenously at a dose of 2.4mg/kg bodyweight on day 0, and after 12 hours.

The vials and ampoules will be stored at room temperature in an air-conditioned room at the study site, with access limited to the investigators only.

4.3.2 Activated Charcoal

Activated charcoal (Aktivkohle, Granulat, 1,5 mm, reinst) is manufactured by Caesar & Loretz GmbH, Germany. It comes as black granules in pre-packaged sachets of 50g.

The 50g AC will be dissolved in 350mls of water and will be given orally to subjects. The granules will be stored at room temperature in an air-conditioned room at the study site, with access limited to the investigators only

5 SELECTION AND WITHDRAWAL OF SUBJECTS

5.1 Selection of Subjects

The study population will be drawn from the villages in the Farafenni area, Upper River Division, The Gambia. It is planned to approach potential volunteers in the field. The investigators and a team of field workers (FW) will go to the community and explain the study during a sensitisation visit. Villages that are interested in the study will be visited by the FW when the trial is ready to start. The information sheet will be made available to those interested in the study. All persons expressing an interest in participating will be invited for screening for which verbal consent will be obtained.

5.2 Eligibility of Subjects

Inclusion Criteria:

Subjects must meet all of the inclusion criteria listed below in order to be eligible to participate in the trial.

- Healthy Gambian adult aged 21 to 45 years of any tribe of African origin.
- No malaria parasites in the blood (demonstrated by a negative optimal test and confirmed on a thick film)

Exclusion Criteria:

All subjects meeting any of the exclusion criteria listed below at baseline will be excluded from trial participation.

- Hb < 11g/dl
- Concurrent participation in any other study
- Breastfeeding or pregnant women
- Taken any medications in the past one week

5.3 Withdrawal of Subjects

Subjects may withdraw voluntarily from participation in the study at any time. Subjects may also withdraw voluntarily from receiving the study intervention for any reason. Subjects who dropped out for any reason will not be replaced.

Subjects who withdrew from the trial will be asked to come for a health check to ensure that the participant is in good health.
Subjects who withdrew from the trial due to an adverse event will be followed until resolution of the symptom or laboratory change occurs, or until a non-study related causality is assigned (see Section 7).

6 TREATMENT OF SUBJECTS

There are no restrictions to other medications besides antimalarial treatment before and during the trial.

All study participants will be given an ITN at the end of the study. For a period of 2 months the study participants and their immediate family (wive(s), husband, biological children) will be granted access to the MRC ward at the hospital. A SEN will accompany the screening team and will be equipped to treat minor acute ailments.

7 TRIAL PROCEDURES AND EVALUATIONS

7.1 Trial Schedule

For an overview see Appendix A: Summary Participant Schedule

7.1.1 Screening

Screening will be performed in the field. It will consist of a finger prick sample to estimate Haemoglobin by (**HemoCue®**) and to look for malaria parasites using Malaria antigen test (**OptiMAL®**). A slide with a thick film will be prepared for microscopic exclusion of malaria parasites. Only slides from those who were found eligible and are willing to participate will be examined on the MRC ward at AFPRC hospital.
All women of child bearing age will be asked if they are pregnant or breastfeeding. Any woman of child bearing age who has missed her last menstrual period will not undergo any of the screening procedures.

For ease of identification, all volunteers will be given a piece of paper with a unique 3-digit screening number on it to enable identification at the hospital. Volunteers that have been found eligible will be invited to the AFPRC hospital either by 7.00 pm the day of screening or by 7.00 pm of the following day.

For the duration of the study volunteers will be treated as inpatients at the MRC ward in the AFPRC hospital. Written informed consent will be obtained prior to a clinical examination performed by the study physician. All women will undergo a pregnancy test and results must be available prior to administration of artesunate.

7.1.2 Enrolment

Eligible volunteers will be enrolled into the study and allocated to one of the three treatment groups according to randomisation (see section 4.2).

The study physician will perform a clinical assessment. Subjects assessed to be clinically fit will be enrolled in the study and allocated to one of the three treatment groups according to randomisation (see section 4.2).

An indwelling forearm catheter will be placed. The first i.v. blood sample will be taken for routine biochemistry, FBC, and a thick film investigation for malaria parasites.

7.1.3 Follow-up

A physical examination will be performed after 18-24hours at discharge. Any adverse event will be followed until resolution of the symptom or laboratory change occurs, or until a non-study related causality is assigned

7.2 Trial Evaluations

7.2.1 *Clinical Evaluations*

The clinical assessment by the trial physician includes a physical examination to measure weight and the vital signs (axillary body temperature, pulse rate, blood pressure, and respiratory rate). The heart and chest will be auscultated, the liver and spleen will be palpated.

Vital signs will be assessed before application of artesunate and after 12h and 18-24 h.

7.2.2 *Laboratory Evaluations*

A routine biochemistry and hematology will be performed before the application of artesunate.

- Biochemistry:
  potassium, sodium, urea, creatinine, total bilirubin, alkaline phosphatase (ALPH), aspartate aminotransferase (AST), albumin.
- Hematology:
  Hemoglobin, hematocrit, white blood cells (WBC) with differential count, platelet count.

Serum creatinine and albumin will be measured in addition at the same time points when the plasma levels of artesunate and DHA will be determined.

Parasite determination:
Apart from the (**OptiMAL®** test, the thick blood film prepared in the field will be investigated for malaria parasites by an experienced slide reader before application of artesunate

Plasma level determination
Plasma samples will be collected to determine the levels of artesunate and its metabolite DHA as well as para-aminobenzoic acid (PABA) at the time points 5, 10, 15, 30, 60, 90 min and 3 and 6 hours after the second dose of artesunate has been given. Samples will be frozen immediately and stored in LiN2 at the MRC field station nearby, and will be transported on dry ice via Fajara to the London School of Hygiene and Tropical Medicine in the UK. Here, samples will be analyzed by liquid chromatography – mass spectrometry (LC-MS)

8 SAFETY CONSIDERATIONS

All adverse events (AE) occurring in participants after administration of one of the investigational products will be recorded and reported as described in the Standard Operating Procedure “Adverse Events (AE) & Serious Adverse Events (SAE)”.

Every AE observed or reported from the day of the first administration of a study drug (Day 0) will be recorded on the Case Report Form (CRF). All AEs will be followed until resolution of the symptom or laboratory change occurs, or until a non-study related causality is assigned.

The investigator will make interpretation of the causal relationship of the adverse event (AE) in question to the intervention.

All serious adverse events (SAEs) will be reported by telephone, email or fax to the local safety monitor, and the Joint Gambian Government/ MRC Ethics Committee within one working day of the investigator becoming aware of the SAE occurrence. The local safety monitor will review SAEs immediately after they occur and follow these events until resolution.

The local safety monitor, who is independent of the trial, will provide real-time safety oversight. The local safety monitor will review the available safety data and may offer guidance on how to proceed.

9 STATISTICAL CONSIDERATIONS

The statistical methods and analysis will be described in detail in the statistical plan.

The primary endpoint is the area under each subject's artesunate (ARS) concentration-time curve (AUC). Secondary endpoints are a) the maximum concentration (C_{max}), time to reach maximum concentration (T_{max}), and half life ($t_{½}$) for ARS, and b) the above four parameters for DHA.

The null hypothesis for each comparison is that the mean value of the endpoint does not differ between control and intervention arms. The alternative hypothesis is that the mean values of each endpoint do differ between control and intervention. Each alternative hypothesis is two-sided (ie intended to detect superiority or inferiority).

To be included in the analysis, a subject must have missed at most one of the follow-up times. Any subject in either intervention arm who did not receive all two doses of charcoal will not be included in the analysis. Any subject in the control arm who received one or more doses of charcoal will not be included in the analysis.
Following Nealon et al.(14), both one- and two-compartment models will be used for both ARS and DHA, and the better fit model will be used. The packages nlmeODE, odesolve and nlme be used with the R software (23).

Primary analysis will be by one way ANOVA on the log-transformed values of AUC and other pharmacokinetic variables. Each intervention arm will be compared to control by a contrast. If the untransformed variables have a distribution closer to normal than the log-transformed ones, then a secondary analysis will be ANOVA on the untransformed variables.

To achieve 80% power to detect a ratio of means of 1.5 of mean AUC, or other pharmacokinetic parameter, between control and either intervention arm, with coefficient of variation of 50% and two-sided significance level of 5%, we will need 23 subjects per arm (Table 2 of van Belle and Martin, 1993, American Statistician, 47: 165-167). With 30 subjects per arm, we will achieve this sample size if 20% are lost to follow-up or otherwise ineligible for inclusion in the analysis.

10 DIRECT ACCESS TO SOURCE DATA

The investigator will permit monitoring and reviews by the ethics committee and authorized representatives of the medical governmental regulatory authorities providing direct access to source data and source documents for the purposes of quality assurance and evaluation of the study safety and progress.

All protocol required clinical information and source data (e.g., blood results) will be collected in Case Report Forms (CRFs) designed by the investigator, that might serve as source documents.

11 QUALITY CONTROL AND QUALITY ASSURANCE

The trial will be monitored by the internal trial monitor. The trial monitor will perform monitoring site visits before and throughout the trial. The purpose of the visits includes to ensuring protocol adherence, accurate capture of clinical data, and appropriate documentation in the Master File.

12 ETHICS

This protocol will be approved by the joint Gambia Government/MRC Ethics Committee before this trial commences. The trial will be conducted in full conformity with the principles set forth in the current revision of the Declaration of Helsinki, International Ethical Guidelines for Biomedical Research Involving Human Subjects (2002), and the MRC Ethics Guide (2004).

For potential risks and benefits see Section 2.3.

12.1 Informed Consent Process

Oral informed consent will be obtained from all participants prior to screening.

Either the study physician or a field worker will explain the study to the potential volunteer, outlining the potential risks, and will answer questions that may arise. If they agree to take part, written informed consent will be obtained from all participants prior to enrolment. The consent form to be used will have been approved by the ethics committee. In any participant without fluent English language skills, a translation in their own language will be provided. A copy of the informed consent document will be given to the subjects for their records.

The subjects may withdraw consent at any time throughout the course of the trial. The rights and welfare of the infants will be protected by emphasizing to the parents that the quality of their medical care will not be adversely affected if they decline to participate in this study.

12.2 Subject Confidentiality

Blood samples and Case Report Forms will be coded by initials and hospital number only.

Section 1 of the CRF used by the fieldworkers will have the volunteer's personal details, and will be stored together with the signed copies of the consent form separately from the other parts of the CRF, in a locked filing cabinet. Sections 2-3 of the CRF will be identifiable by the study participants ID number only, and will be stored separately in a locked filing cabinet

All records will be kept in a locked filing cabinet, which is accessed only by the listed investigators. All computer entry and networking programs will be done with coded numbers only. Only authorized persons will have access to the data (see section 10)
Signed consent forms will be stored separately from other records in a locked filing cabinet in a secure office.

Every effort will be taken to maintain confidentiality. Clinical information will not be released without

written permission of the participants with the exceptions stated in Section 10.

12.3 Biohazard Containment

As the transmission of HIV and other blood-borne pathogens can occur through contact with contaminated needles, blood, and blood products, appropriate blood and secretion precautions will be employed by all personnel in the drawing of blood and shipping and handling of all specimens for this study, according to the MRC safety manual.

13 DATA HANDLING AND RECORD KEEPING

The investigators will maintain appropriate medical and research records for this trial. The investigator is responsible to ensure the accuracy, completeness, legibility, and timeliness of the data reported. All source documents should be completed in a neat, legible manner to ensure accurate interpretation of data.

All data on the Case Report Forms must be legibly recorded in blue or black ink or typed. A correction should be made by striking through the incorrect entry with a single line and entering the correct information adjacent to it. The correction must be initialed and dated by the investigator or a designated, qualified individual.

Any requested information that is not obtained as specified in the protocol should have an explanation noted on the CRF as to why the required information was not obtained.
All source documents and laboratory reports must be reviewed by the clinical team and data entry staff, who will ensure that they are accurate and complete.

For confidentiality of data see Section 12.2.

The data manager will be responsible for receiving, entering, cleaning, querying, analyzing and storing all data that accrues from the trial.

Clinical data including AEs and clinical laboratory data will be entered into Microsoft Access. The data system includes password protection and internal quality checks, such as automatic range checks, to identify data that appear inconsistent, incomplete, or inaccurate. Clinical data will be entered directly from the source documents.

Data for this trial will include laboratory and safety measures.

All records pertaining to this study will be kept for a period of at least 10 years before destruction.

Protocol Deviations
The investigator will conduct the trial in compliance with the protocol which was given approval by the ethics committee.

The investigator will not implement any deviation from or changes of the protocol without prior review and documented approval from the ethics committee of an amendment, except where necessary to eliminate an immediate hazard(s) to trial subjects, or when the change(s) involve only logistical or administrative aspects of the trial (e.g., change in monitor(s), change of telephone number(s)).

The investigator, or person designated by the investigator, will document and explain any deviation from the approved protocol in the protocol deviation file.

A protocol deviation is any noncompliance with the clinical trial protocol, Good Clinical Practice (GCP), or other defined procedures requirements. The noncompliance may be either on the part of the subject, the investigator, or the study site staff. As a result of deviations, corrective actions are to be developed by the site and implemented promptly.

The investigator may implement a deviation from or a change of the protocol to eliminate an immediate hazard(s) to trial subjects without prior ethics approval. As soon as possible, the implemented deviation or change, the reasons for it, and, if appropriate, the proposed protocol amendment(s) should be submitted to the ethics committee for review and approval.

It is the responsibility of the site to use continuous vigilance to identify and report deviations.

14 INDEMNITY

In case of any harm as a direct result of participating in the trial medical care and treatment will be provided for free.

15 PLANS FOR DISTRIBUTION OF RESEARCH FINDINGS

At the end of the study, a research report of the methods, detailed results, and brief conclusions will be prepared. These data are a prerequisite for the design of further studies that aim to explore the efficacy of such an approach in mild cases of malaria, and eventually in severely ill patients.

Unit seminars will be given, and data may be presented on scientific conferences.

16 LITERATURE REFERENCES

1. **Bryce, J., C. Boschi-Pinto, K. Shibuya, and R. E. Black. 2005. WHO estimates of the causes of death in children. *Lancet 365:1147*.**

2. **Wang, H., O. Bloom, M. Zhang, J. M. Vishnubhakat, M. Ombrellino, J. Che, A. Frazier, H. Yang, S. Ivanova, L. Borovikova, K. R. Manogue, E. Faist, E. Abraham, J. Andersson, U. Andersson, P. E. Molina, N. N. Abumrad, A. Sama, and K. J. Tracey. 1999. HMG-1 as a late mediator of endotoxin lethality in mice. *Science 285:248*.**

3. **Czura, C. J., H. Yang, C. A. Amella, and K. J. Tracey. 2004. HMGB1 in the immunology of sepsis (not septic shock) and arthritis. *Adv Immunol 84:181*.**

4. **Ulloa, L., M. Ochani, H. Yang, M. Tanovic, D. Halperin, R. Yang, C. J. Czura, M. P. Fink, and K. J. Tracey. 2002. Ethyl pyruvate prevents lethality in mice with established lethal sepsis and systemic inflammation. *Proc Natl Acad Sci U S A 99:12351*.**

5. **Wang, H., H. Liao, M. Ochani, M. Justiniani, X. Lin, L. Yang, Y. Al-Abed, C. Metz, E. J. Miller, K. J. Tracey, and L. Ulloa. 2004. Cholinergic agonists inhibit HMGB1 release and improve survival in experimental sepsis. *Nat Med 10:1216*.**

6. **1999. Position statement and practice guidelines on the use of multi-dose activated charcoal in the treatment of acute poisoning. American Academy of Clinical Toxicology; European Association of Poisons Centres and Clinical Toxicologists. *J Toxicol Clin Toxicol 37:731*.**

7. Bradberry, S. M., and J. A. Vale. 1995. Multiple-dose activated charcoal: a review of relevant clinical studies. *J Toxicol Clin Toxicol 33:407*.

8. Schulman, G. 2006. A nexus of progression of chronic kidney disease: charcoal, tryptophan and profibrotic cytokines. *Blood Purif 24:143*.

9. Akintonwa, A., and O. E. Orisakwe. 1990. The adsorption of quinine and quinidine to activated charcoal with and without magnesium sulfate. *Vet Hum Toxicol 32:567*.

10. Lockey, D., and D. N. Bateman. 1989. Effect of oral activated charcoal on quinine elimination. *Br J Clin Pharmacol 27:92*.

11. Prescott, L. F., A. R. Hamilton, and R. Heyworth. 1989. Treatment of quinine overdosage with repeated oral charcoal. *Br J Clin Pharmacol 27:95*.

12. Hasan, M. M., M. A. Hassan, and N. M. Rawashdeh. 1990. Effect of oral activated charcoal on the pharmacokinetics of quinidine and quinine administered intravenously to rabbits. *Pharmacol Toxicol 67:73*.

13. Dondorp, A., F. Nosten, K. Stepniewska, N. Day, and N. White. 2005. Artesunate versus quinine for treatment of severe falciparum malaria: a randomised trial. *Lancet 366:717*.

14. Nealon, C., A. Dzeing, U. Muller-Romer, T. Planche, V. Sinou, M. Kombila, P. G. Kremsner, D. Parzy, and S. Krishna. 2002. Intramuscular bioavailability and clinical efficacy of artesunate in gabonese children with severe malaria. *Antimicrob Agents Chemother 46:3933*.

15. Davis, T. M., H. L. Phuong, K. F. Ilett, N. C. Hung, K. T. Batty, V. D. Phuong, S. M. Powell, H. V. Thien, and T. Q. Binh. 2001. Pharmacokinetics and pharmacodynamics of intravenous artesunate in severe falciparum malaria. *Antimicrob Agents Chemother 45:181*.

16. Batty, K. T., L. T. Thu, T. M. Davis, K. F. Ilett, T. X. Mai, N. C. Hung, N. P. Tien, S. M. Powell, H. V. Thien, T. Q. Binh, and N. V. Kim. 1998. A pharmacokinetic and pharmacodynamic study of intravenous vs oral artesunate in uncomplicated falciparum malaria. *Br J Clin Pharmacol 45:123*.

17. Genovese, R. F., D. B. Newman, J. M. Petras, and T. G. Brewer. 1998. Behavioral and neural toxicity of arteether in rats. *Pharmacol Biochem Behav 60:449*.

18. Brewer, T. G., S. J. Grate, J. O. Peggins, P. J. Weina, J. M. Petras, B. S. Levine, M. H. Heiffer, and B. G. Schuster. 1994. Fatal neurotoxicity of arteether and artemether. *Am J Trop Med Hyg 51:251*.

19. Kissinger, E., T. T. Hien, N. T. Hung, N. D. Nam, N. L. Tuyen, B. V. Dinh, C. Mann, N. H. Phu, P. P. Loc, J. A. Simpson, N. J. White, and J. J. Farrar. 2000. Clinical and

neurophysiological study of the effects of multiple doses of artemisinin on brain-stem function in Vietnamese patients. *Am J Trop Med Hyg 63:48*.

20. **Nontprasert, A., M. Nosten-Bertrand, S. Pukrittayakamee, S. Vanijanonta, B. J. Angus, and N. J. White. 1998. Assessment of the neurotoxicity of parenteral artemisinin derivatives in mice. *Am J Trop Med Hyg 59:519*.**

21. **Price, R., M. van Vugt, L. Phaipun, C. Luxemburger, J. Simpson, R. McGready, F. ter Kuile, A. Kham, T. Chongsuphajaisiddhi, N. J. White, and F. Nosten. 1999. Adverse effects in patients with acute falciparum malaria treated with artemisinin derivatives. *Am J Trop Med Hyg 60:547*.**

22. **Davis, T. M., T. Q. Binh, K. F. Ilett, K. T. Batty, H. L. Phuong, G. M. Chiswell, V. D. Phuong, and C. Agus. 2003. Penetration of dihydroartemisinin into cerebrospinal fluid after administration of intravenous artesunate in severe falciparum malaria. *Antimicrob Agents Chemother 47:368*.**

23. **Tornoe, C. W., H. Agerso, E. N. Jonsson, H. Madsen, and H. A. Nielsen. 2004. Non-linear mixed-effects pharmacokinetic/pharmacodynamic modelling in NLME using differential equations. *Comput Methods Programs Biomed 76:31*.**

APPENDIX A: SUMMARY PARTICIPANT SCHEDULE

Time	Procedure
**0h – evening day 0**<br>**(Time point 1)**	- Clinical assessment by qualified physician<br>-FBC, Full Biochemistry, Thick film<br>- IV artesunate to all subjects<br>- Plain water to subjects in group 1<br>- AC to subjects in group 2 only,
**1h ± 5min***<br>**(Time point 1b)**	- AC to subjects in group 3 only
**12h ± 5min***<br>**(Time point 2)**	- Clinical assessment by qualified physician<br>- 2nd dose of IV artesunate to all subjects<br>- Plain water to subjects in group 1<br>- AC to subjects in group 2 only
**12h 5 min***<br>**(Time point 3)**	- Serum Creatinine, Albumin<br>- Plasma Artesunate, DHA
**12h 10 min***<br>**(Time point 4)**	- Serum Creatinine, Albumin.<br>- Plasma Artesunate, DHA
**12h 15 min***<br>**(Time point 5)**	- Serum Creatinine, Albumin.<br>- Plasma Artesunate, DHA
**12h 30 min***<br>**(Time point 6)**	- Serum Creatinine, Albumin.<br>- Plasma Artesunate, DHA
**13h ± 5min***<br>**(Time point 7)**	- Serum Creatinine, Albumin.<br>- Plasma Artesunate, DHA<br>- AC to subjects in group 3 only
**13h 30 min ± 5min***<br>**(Time point 8)**	- Serum Creatinine, Albumin.<br>- Plasma Artesunate, DHA
**15h ± 5min***<br>**(Time point 9)**	- Serum Creatinine, Albumin.<br>- Plasma Artesunate, DHA
**18h ± 5min***<br>**(Time point 10)**	- Full biochemistry<br>- Plasma Artesunate, DHA
**18h 5min – 24hrs**	- Clinical assessment by qualified physician<br>- Discharge

* All time points measured from the time of the first dose of IV artesunate.

For details see Section 7.
