# Supplementary material for: Oral Activated Charcoal Prevents Experimental Cerebral Malaria in Mice and in a Randomized Controlled Clinical Trial in Man Did Not Interfere with the Pharmacokinetics of Parenteral Artesunate
Source: PLoS One. 2010 Apr 15;5(4):e9867. doi: 10.1371/journal.pone.0009867 (PMC2855344; doi:10.1371/journal.pone.0009867)
Supplement: Table S1 — Characteristics of the study population according to study group. (0.03 MB DOC) [file pone.0009867.s003.doc]

| **Arm** | **1** | **2** | **3** |
| --- | --- | --- | --- |
| **Weight (kg)** | 59 (55 - 64) | 62 (58 - 67) | 60 (56 - 65) |
| **Dose (mg)** | 142 (132 - 154) | 149 (139 – 161) | 144 (135 - 155) |
| **Age (years)** | 34 (30 - 38) | 38 (34 - 41) | 35 (31 - 38) |
| **Male participants (%)** | 64.7 | 72.2 | 62.5 |

**Supplementary Table 1.**  **Characteristics of the study population according to study group.**

For age, weight and dose medians (with 95% CI) are shown.
